# The Preventable Causes of Death in the United States: Comparative Risk Assessment of Dietary, Lifestyle, and Metabolic Risk Factors

**DOI:** 10.1371/journal.pmed.1000058

**Published:** 2009-04-28

**Authors:** Goodarz Danaei, Eric L. Ding, Dariush Mozaffarian, Ben Taylor, Jürgen Rehm, Christopher J. L. Murray, Majid Ezzati

**Affiliations:** 1 Harvard School of Public Health, Boston, Massachusetts, United States of America; 2 Initiative for Global Health, Harvard University, Cambridge, Massachusetts, United States of America; 3 Harvard Medical School, Boston, Massachusetts, United States of America; 4 Centre for Addiction and Mental Health, University of Toronto, Toronto, Canada; 5 Public Health Sciences, University of Toronto, Toronto, Canada; 6 Clinical Psychology and Psychotherapy, Technische Universität Dresden, Dresden, Germany; 7 Institute for Health Metrics and Evaluation, The University of Washington, Seattle, Washington, United States of America; University of Otago, New Zealand

## Abstract

**Background:**

Knowledge of the number of deaths caused by risk factors is needed for health policy and priority setting. Our aim was to estimate the mortality effects of the following 12 modifiable dietary, lifestyle, and metabolic risk factors in the United States (US) using consistent and comparable methods: high blood glucose, low-density lipoprotein (LDL) cholesterol, and blood pressure; overweight–obesity; high dietary trans fatty acids and salt; low dietary polyunsaturated fatty acids, omega-3 fatty acids (seafood), and fruits and vegetables; physical inactivity; alcohol use; and tobacco smoking.

**Methods and Findings:**

We used data on risk factor exposures in the US population from nationally representative health surveys and disease-specific mortality statistics from the National Center for Health Statistics. We obtained the etiological effects of risk factors on disease-specific mortality, by age, from systematic reviews and meta-analyses of epidemiological studies that had adjusted (i) for major potential confounders, and (ii) where possible for regression dilution bias. We estimated the number of disease-specific deaths attributable to all non-optimal levels of each risk factor exposure, by age and sex. In 2005, tobacco smoking and high blood pressure were responsible for an estimated 467,000 (95% confidence interval [CI] 436,000–500,000) and 395,000 (372,000–414,000) deaths, accounting for about one in five or six deaths in US adults. Overweight–obesity (216,000; 188,000–237,000) and physical inactivity (191,000; 164,000–222,000) were each responsible for nearly 1 in 10 deaths. High dietary salt (102,000; 97,000–107,000), low dietary omega-3 fatty acids (84,000; 72,000–96,000), and high dietary trans fatty acids (82,000; 63,000–97,000) were the dietary risks with the largest mortality effects. Although 26,000 (23,000–40,000) deaths from ischemic heart disease, ischemic stroke, and diabetes were averted by current alcohol use, they were outweighed by 90,000 (88,000–94,000) deaths from other cardiovascular diseases, cancers, liver cirrhosis, pancreatitis, alcohol use disorders, road traffic and other injuries, and violence.

**Conclusions:**

Smoking and high blood pressure, which both have effective interventions, are responsible for the largest number of deaths in the US. Other dietary, lifestyle, and metabolic risk factors for chronic diseases also cause a substantial number of deaths in the US.

*Please see later in the article for Editors' Summary*

## Abstract 2

Majid Ezzati and colleagues examine US data on risk factor exposures and disease-specific mortality and find that smoking and hypertension, which both have effective interventions, are responsible for the largest number of deaths.

## Editors' Summary

**Background:**

A number of modifiable factors are responsible for many premature or preventable deaths. For example, being overweight or obese shortens life expectancy, while half of all long-term tobacco smokers in Western populations will die prematurely from a disease directly related to smoking. Modifiable risk factors fall into three main groups. First, there are lifestyle risk factors. These include tobacco smoking, physical inactivity, and excessive alcohol use (small amounts of alcohol may actually prevent diabetes and some types of heart disease and stroke). Second, there are dietary risk factors such as a high salt intake and a low intake of fruits and vegetables. Finally, there are “metabolic risk factors,” which shorten life expectancy by increasing a person's chances of developing cardiovascular disease (in particular, heart problems and strokes) and diabetes. Metabolic risk factors include having high blood pressure or blood cholesterol and being overweight or obese.

**Why Was This Study Done?:**

It should be possible to reduce preventable deaths by changing modifiable risk factors through introducing public health policies, programs and regulations that reduce exposures to these risk factors. However, it is important to know how many deaths are caused by each risk factor before developing policies and programs that aim to improve a nation's health. Although previous studies have provided some information on the numbers of premature deaths caused by modifiable risk factors, there are two problems with these studies. First, they have not used consistent and comparable methods to estimate the number of deaths attributable to different risk factors. Second, they have rarely considered the effects of dietary and metabolic risk factors. In this new study, the researchers estimate the number of deaths due to 12 different modifiable dietary, lifestyle, and metabolic risk factors for the United States population. They use a method called “comparative risk assessment.” This approach estimates the number of deaths that would be prevented if current distributions of risk factor exposures were changed to hypothetical optimal distributions.

**What Did the Researchers Do and Find?:**

The researchers extracted data on exposures to these 12 selected risk factors from US national health surveys, and they obtained information on deaths from difference diseases for 2005 from the US National Center for Health Statistics. They used previously published studies to estimate how much each risk factor increases the risk of death from each disease. The researchers then used a mathematical formula to estimate the numbers of deaths caused by each risk factor. Of the 2.5 million US deaths in 2005, they estimate that nearly half a million were associated with tobacco smoking and about 400,000 were associated with high blood pressure. These two risk factors therefore each accounted for about 1 in 5 deaths in US adults. Overweight–obesity and physical inactivity were each responsible for nearly 1 in 10 deaths. Among the dietary factors examined, high dietary salt intake had the largest effect, being responsible for 4% of deaths in adults. Finally, while alcohol use prevented 26,000 deaths from ischemic heart disease, ischemic stroke, and diabetes, the researchers estimate that it caused 90,000 deaths from other types of cardiovascular diseases, other medical conditions, and road traffic accidents and violence.

**What Do These Findings Mean?:**

These findings indicate that smoking and high blood pressure are responsible for the largest number of preventable deaths in the US, but that several other modifiable risk factors also cause many deaths. Although the accuracy of some of the estimates obtained in this study will be affected by the quality of the data used, these findings suggest that targeting a handful of risk factors could greatly reduce premature mortality in the US. The findings might also apply to other countries, although the risk factors responsible for most preventable deaths may vary between countries. Importantly, effective individual-level and population-wide interventions are already available to reduce people's exposure to the two risk factors responsible for most preventable deaths in the US. The researchers also suggest that combinations of regulation, pricing, and education have the potential to reduce the exposure of US residents to other risk factors that are likely to shorten their lives.

**Additional Information:**

Please access these Web sites via the online version of this summary at http://dx.doi.org/10.1371/journal.pmed.1000058.

- The MedlinePlus encyclopedia contains a page on healthy living (in English and Spanish)
- The US Centers for Disease Control and Prevention provides information on all aspects of healthy living
- Healthy People 2010 is a national framework designed to improve the health of people living in the US. The Healthy People 2020 Framework is due to be launched in January 2010
- The World Health Report 2002Reducing Risks, Promoting Healthy Life provides a global analysis of how healthy life expectancy could be increased
- The National Health and Nutrition Examination Survey (NHANES) is “a program of studies designed to assess the health and nutritional status of adults and children in the United States”
- The US Centers for Disease Control and Prevention's site Smoking and Tobacco Use offers a large number of informational and data resources on this important risk factor
- The American Heart Association and American Cancer Society provide a rich resource for patients and caregivers on many important risk factors including diet, sodium intake, and smoking

## Introduction

Valid and comparable information on mortality caused by diseases, injuries, and their modifiable risk factors is important for health policy and priority setting [1],[2]. The standard death certificate is valuable for assigning deaths to specific diseases or injuries, but does not provide information on the modifiable risk factors that cause these diseases. Previous research has indicated that modifiable risk factors are responsible for a large number of premature deaths in the United States [1],[3]. However, prior analyses did not use consistent and comparable methods for the mortality effects of different risk factors. More importantly, previous analyses did not include any dietary risk factors. The only metabolic risk factor—i.e., those measured by physiological indicators such as blood pressure, blood glucose, serum cholesterol, and body mass index (BMI)—in these analyses was overweight–obesity.

We estimated the number of deaths attributable to major dietary, lifestyle, and metabolic risk factors in the US using consistent, comparable, and current definitions, methods, and data sources. We conducted the analysis in the US because the results can inform priority-setting decisions for policies and programs that aim to improve the nation's health, e.g., *Healthy People 2010* and (the forthcoming) *Healthy People 2020*. The US also has high-quality data on disease-specific mortality and on population exposure to a range of risk factors from nationally representative health examination and interview surveys. Our results provide, to our knowledge, the most comprehensive and comparable quantitative assessment of the mortality burden of important modifiable risk factors in the US population, and the only one to include the effects of multiple dietary and metabolic factors.

## Methods

We conducted a population-level CRA (comparative risk assessment) for 12 major modifiable dietary, lifestyle, and metabolic risks. The CRA analysis estimates the number of deaths that would be prevented in the period of analysis if current distributions of risk factor exposure were changed to a hypothetical alternative distribution. The inputs to the analysis are (1) the current population distribution of risk factor exposure, (2) the etiological effect of risk factor exposures on disease-specific mortality, (3) an alternative exposure distribution, and (4) the total number of disease-specific deaths in the population.

**Table 1:** Risk factors in this analysis, their exposure variables, theoretical-minimum-risk exposure distributions, disease outcomes, and data sources for exposure.

Risk Factor	Exposure Metric	Exposure Data Sources	TMRED±SD	Disease Outcomes^a^
**High blood glucose**	Usual level of fasting plasma glucose [61]	NHANES 2003–2006 (SD corrected for intra-individual variation)	4.9±0.3 mmol/l [61]	IHD; stroke; renal failure; *colorectal, breast, and pancreatic cancers*
**High LDL cholesterol** b	Usual level of LDL cholesterol	NHANES 2003–2006 (SD corrected for intra-individual variation)	2.0±0.44 mmol/l^c^ [62]	IHD; ischemic stroke; *selected other cardiovascular diseases*
**High blood pressure**	Usual level of systolic blood pressure	NHANES 2003–2006 (SD corrected for intra-individual variation)	115±6 mmHg [63], [64]	IHD, stroke, hypertensivedisease, other cardiovascular diseases^d^, *renal failure*
**Overweight–obesity (high BMI)**	BMI	NHANES 2003–2006	21±1 kg/m^2^ [21], [65]	IHD; ischemic stroke; hypertensive disease; diabetes mellitus; corpus uteri, colon, kidney, and postmenopausal breast cancers; *gallbladder cancer* e
**High dietary trans fatty acids**	Usual percent of total calories from dietary trans fatty acids	CSFII 1989–1991^f^	0.5%±0.05% of total calories from trans fatty acids [16]	IHD
**Low dietary poly-unsaturated fatty acids (PUFA) (in replacement of saturated fatty acids; see ** **Table 2** **)**	Usual percent of total calories from dietary PUFA	NHANES 2003–2006	10%±1% of total calories from PUFA	IHD, stroke
**Low dietary omega-3 fatty acids (seafood)**	Usual dietary omega-3 fatty acids in five categories adjusted for total calories^g^	NHANES 2003–2006	250 mg/d [31]	IHD, stroke
**High dietary salt (sodium)** h	Usual level of dietary sodium adjusted for total calories	NHANES 2003–2006	0.5±0.05 g/d [66]	IHD, stroke, hypertensivedisease, other cardiovascular diseases, stomach cancer, *renal failure*
**Low intake of fruits and vegetables**	Usual dietary fruit and vegetable intake adjusted for total calories^i^	NHANES 2003–2006	600±50 g/d [67]	IHD; ischemic stroke; colorectal, stomach, lung, esophagus, mouth, and pharyngeal cancers
**Alcohol use**	Current alcohol consumption volumes and patterns^j^; prevalence of alcohol use among emergency room patients; BAC levels of drivers in road traffic injuries	NESARC 2001–2002, FARS 2005 and emergency room studies	No alcohol use^k^	IHD; ischemic stroke; hemorrhagic stroke; hypertensive disease; cardiac arrhythmias; diabetes mellitus; liver, mouth, and pharynx, larynx, breast, esophagus, colorectal, selected other cancers^l^; liver cirrhosis; acute and chronic pancreatitis; road traffic injuries; falls; homicide and suicide; other injuries; alcohol use disorders^m^; *selected other cardiovascular diseases; hepatitis C; epilepsy; fetal effects of alcohol use during pregnancy; tuberculosis*
**Physical inactivity**	Physical activity measured in four categories: inactive, low-active, moderately active, and highly active^n^	NHANES 2003–2006	The whole population being highly active (≥1 h/wk of vigorous activity and at least 1,600 met·min/wk)^o^	IHD; ischemic stroke; breast cancer and colon cancers; diabetes mellitus
**Tobacco smoking**	Current levels of Smoking Impact Ratio (SIR) (indirect indicator of accumulated smoking risk based on excess lung cancer mortality) [18] p	Lung cancer mortality from adjusted vital registration in 2004	No smoking	IHD; stroke; selected other cardiovascular diseases; diabetes mellitus; lung, esophagus, mouth and pharynx, stomach, liver, pancreas, cervix, bladder, kidney and other urinary cancers; leukemia; chronic obstructive pulmonary disease (COPD); other respiratory diseases^q^ tuberculosis; *colorectal cancer and hypertensive disease* r, *burns and fire injuries, effects of smoking during pregnancy on maternal and perinatal conditions*

a Outcomes in italics are those for which the effects were not quantified in the main analysis due to weaker evidence on causality (e.g. tobacco smoking and colorectal cancer or high blood glucose and cancers) or because there were very few deaths from the disease (e.g. high BMI and gallbladder cancer).

b We evaluated sensitivity to the choice of exposure metric by using total cholesterol instead of LDL-cholesterol (Table S1).

c Two alternative TMREDs for LDL cholesterol with means of 1.6 mmol/l and 2.3 mmol/l were examined in sensitivity analysis (Table S1).

d This category includes rheumatic heart disease, acute and subacute endocarditis, cardiomyopathy, other inflammatory cardiac diseases, valvular disorders, aortic aneurysm, pulmonary embolism, conduction disorders, peripheral vascular disorders, and other ill-defined cardiovascular diseases.

e We did not include some of the cancers that were found to have significant association with BMI in a recent meta-analysis [17] either because there were very few deaths in the US (adenocarcinoma of esophagus and gallbladder cancer) or because there was not strong evidence on a causal effect from other studies (leukemia and multiple myeloma). We included non-Hodgkin lymphoma in a sensitivity analysis (Table S1).

f The NHANES rounds in 2003–2006 include a 2-d dietary intake survey and could be used to estimate dietary trans fatty acids. However, a reliable source for the trans fat content of each food item was not available to us. We have used the intake estimates in the Continuing Survey of Food Intakes by Individuals (CSFII) 1989–1991 [68] in our analysis.

g Omega-3 intake categories in the analysis were: 0 to <62.5; 62.5 to <125; 125 to <187.5; 187.5 to <250; and ≥250 mg/d of eicosapentaenoic acid (EPA) and docosahexaenoic acid (DHA).

h The effect of reduction in salt intake on SBP and the effect of subsequent decline in SBP on the relevant disease outcomes were estimated at the individual level to account for possible correlation between salt intake and SBP.

i We evaluated sensitivity to the assumption of normal distribution for fruit and vegetable intake (Table S1).

j Exposure categories were: Abstainer, a person not having had a drink containing alcohol within the last year; DI, 0–19.99 g of pure alcohol daily (females) and 0–39.99 g (males); DII, 20–39.99 g (females) and 40–59.99 g (males); and DIII, >40 g (females) and >60 g (males). Binge drinking was defined as having at least one occasion of five or more drinks in the last month.

k An alternative TMRED for alcohol use as regular drinking of small amounts of alcohol is considered in sensitivity analysis (Table S1).

l This category includes ICD-9 codes 210–239.

m This category includes ICD-9 codes 291, 303, and 305.0.

n Categories of physical activity were defined as below using responses to questions regarding physical activity during the past 30 d: inactive, no moderate or vigorous physical activity; low-active, <2.5 h/wk of moderate activity or <600 met·min/wk; moderately active, either ≥2.5 h/wk of moderate activity or ≥1 h of vigorous activity and ≥600 met·min/wk; highly active, ≥1 h/wk of vigorous activity and ≥1,600 met·min/wk.

o This TMRED is based on multiple prospective studies that report beneficial effects of physical activity continuing above the current recommended levels [69]–[72].

p We also calculated the mortality effects of tobacco smoking using the prevalence of current and former smokers, as used by Smoking-Attributable Mortality, Morbidity, and Economic Costs (SAMMEC; http://apps.nccd.cdc.gov/sammec) [73], in a sensitivity analysis (Table S1).

q This category includes lower respiratory tract infections and asthma.

r Evidence of a causal association between tobacco smoking and colorectal cancer was classified as suggestive in the 2004 Report of the US Surgeon General [73]. The 2004 report also excluded hypertensive disease from the outcomes considered in smoking-attributable mortality. Therefore, colorectal cancer and hypertensive disease were not included in the main analysis, but were included in sensitivity analysis (Table S1).

We also calculated the proportion of the mortality burden of risk factors among people in specific exposure ranges and categories that correspond to current clinical and public health guidelines, e.g., the proportion of deaths attributable to high blood pressure, that is, among individuals with systolic blood pressure (SBP) ≥140 mmHg. This threshold-based analysis helps evaluate the relative impact of programs that focus on people whose high-risk status is either undiagnosed or remains uncontrolled after receiving the currently administered usual care.

### Selection of Risk Factors

Among dietary, lifestyle, and metabolic factors, we selected specific risks that fulfilled the following criteria. (1) Sufficient evidence was available on the presence and magnitude of likely causal associations with disease-specific mortality from high-quality epidemiological studies; (2) available or envisionable interventions existed to modify exposure to the risk; and (3) data on risk factor exposure were available from nationally representative surveys and epidemiological studies without systematic bias.

The 12 major modifiable risk factors selected based on these criteria are shown in Table 1. High blood pressure and high low-density lipoprotein (LDL) cholesterol were selected as major modifiable risk factors for cardiovascular mortality, with their effects on cardiovascular diseases established in observational as well as randomized studies. High blood glucose, overweight–obesity (high BMI), physical inactivity, five dietary factors, alcohol use, and tobacco smoking were selected as major modifiable risk factors for cardiovascular diseases, cancers, and other diseases. The mortality-reducing effects of omega-3 fatty acids and of replacing saturated fatty acids (SFA) with polyunsaturated fatty acids (PUFA) (denoted as PUFA and PUFA-SFA replacement interchangeably hereafter) have been confirmed in randomized trials. The mortality effects of other risk factors were considered probable or convincing based on the breadth and consistency of evidence from well-conducted observational studies. The relationship between dietary salt (sodium) and cardiovascular mortality was based on convincing effects on blood pressure in intervention studies, as well as on disease outcomes in at least one study.

Several other important risk factors were considered, but could not be included because sufficient or unbiased data on their national exposure distributions and/or effects on disease-specific mortality were not available, or because the evidence on causal effects was less convincing. Examples of important risk factors not included in this work because of insufficient data on exposure or on the presence and magnitude of causal effects include illicit drug use, caloric intake, triglycerides and high-density lipoprotein (HDL) cholesterol, whole-grain intake, gun ownership, and risk factors that primarily affect mental health outcomes. The focus of our analysis was not on environmental and occupational risks (e.g., exposure to urban particle pollution, radon, and arsenic), primarily because for many of these risks nationally representative data on population exposure using the same metrics as used in epidemiological studies are unavailable. Comparative analyses of these risks should be a priority for future research.

### Data Sources

We obtained risk factor exposure distributions from nationally representative health examination and interview surveys, etiological effect sizes from published or new systematic reviews and meta-analyses of epidemiological studies, and the number of deaths by cause from the National Center for Health Statistics (NCHS).

#### Risk factor exposure

For most risk factors in this analysis, we used data from the National Health and Nutrition Examination Survey (NHANES) to measure population exposures (Table 1). NHANES uses a complex multi-stage, stratified, clustered probability sample design to provide nationally representative data on health and nutrition characteristics of the civilian, noninstitutionalized US population. NHANES includes an in-person interview and a subsequent examination component in a MEC (mobile examination clinic); those unable to visit the MEC are offered a limited examination at home. We used two NHANES rounds that covered 2003–2006 to provide sufficient sample size for exposure estimates by age and sex, for years as close as possible to the latest year for which mortality data were available (2005). The total sample size for the 2003–2006 rounds was 20,470. Additional information on survey design and methods is available at http://www.cdc.gov/nchs/nhanes.htm. We adjusted the intakes of fruits and vegetables, omega-3 fatty acids, and salt (sodium) from NHANES for total energy intake using the residual method [4].

We used the National Epidemiologic Survey on Alcohol and Related Conditions (NESARC) to measure the quantity of alcohol consumed and the pattern of consumption. NESARC is a multi-stage, stratified, cluster-sampling, nationally representative survey of the noninstitutionalized US population, whose target population includes boarding houses, rooming houses, nontransient hotels and motels, shelters, facilities for housing workers, college quarters, and group homes. The 2001–2002 sample size was 43,093. Detailed information on frequency, amount, and type of beverage during the previous 12 mo was sought using computer assisted personal interviewing. Average daily alcohol consumption in NESARC was estimated using responses to questions on both the usual quantity-and-frequency of drinking, and quantity-and-frequency of binge drinking. This method provides a more valid estimate of average consumption than do calculations based solely on questions regarding usual drinking [5]. A summary is available online at http://www.census.gov/rophi/www/nesarc.html.

We accounted for complex survey design and sampling weights in estimating exposures. Using one-off measurements in health examination surveys overestimates the standard deviation (SD) of the “usual” population exposure distribution, due to within-person variation. We estimated the usual population SD of blood pressure, fasting plasma glucose (FPG), and LDL cholesterol by multiplying the SD of NHANES sample by the dilution ratio from studies that had multiple exposure measurements [6]–[8]. We did not adjust the SD of BMI for within-person variations in body weight, because studies with multiple BMI measurements have not found evidence for substantial within-person variability in BMI [9]. For dietary factors, we calculated the SD of population exposure by partitioning the within- and between-person variabilities of the two 24-h diet recalls using a random-effect regression model (using XTREG in STATA software). We also did not adjust the distributions of alcohol use and physical activity, because there were very few prospective studies with multiple measurements to provide reliable evidence for the relationship between one-off and usual exposure distributions [9], [10].

#### Etiological effects of risk factors on disease-specific mortality

We obtained the relative risk (RR) per unit of exposure (for risks measured continuously) or for each exposure category (for risks measured in categories) for diseases with probable or convincing causal associations with each risk factor, based on the most recent published systematic reviews and meta-analyses of epidemiological studies or by conducting new systematic reviews and meta-analyses when they were not available in the published literature (Tables 2–7).

**Table 2:** Sources and magnitudes of relative risks for the effects of continuous dietary risk factors on disease-specific mortality.

Risk Factor	Disease Outcome	Source of RR	Units	Age Group	RR
**High dietary trans fatty acids**	IHD^a^	Meta-analysis of three prospective cohort studies [16]	Per one percentage point more calories	30–44	1.40
				45–59	1.29
				60–69	1.14
				70–79	1.08
				80+	1.06
**Low dietary PUFA (in replacement of SFA)**	IHD^a^	Meta-analysis of seven intervention studies by authors^b^	Per one percentage point less calories from PUFA, in isocaloric exchange for SFA^b^	30–44	1.05
				45–59	1.04
				60–69	1.02
				70–79	1.01
				80+	1.01
**High dietary salt**	SBP	Meta-analysis of dietary trials	mmHg SBP per 100 mmol/d dietary sodium	SBP≥140 mmHg	7.11
				SBP<140 mmHg	3.57
	Stomach cancer	Meta-analysis of three prospective cohort studies [74]	Per 100 mmol/d dietary sodium	—	1.57
**Low intake of fruits and vegetables**	IHD	Meta-analysis of six prospective cohort studies [75]	Per 80 g/d lower intake	30–69	1.04
				70–79	1.03
				80+	1.02
	Ischemic stroke	Meta-analysis of three prospective cohort studies [76]	Per 80 g/d lower intake	30–69	1.06
				70–79	1.05
				80+	1.03
	Lung cancer	Meta-analysis of major observational studies [67]	Per 80 g/d lower intake	30–69	1.04
				70–79	1.03
				80+	1.02
	Stomach cancer	Meta-analysis of major observational studies [67]	Per 80 g/d lower intake	30–69	1.06
				70–79	1.05
				80+	1.03
	Colorectal cancer	Meta-analysis of major observational studies [67]	Per 80 g/d lower intake	30–69	1.01
				70–79	1.01
				80+	1.00
	Esophagus, mouth, and pharynx cancers	Meta-analysis of major observational studies [77]	Per 80 g/d lower intake	30–69	1.10
				70–79	1.08
				80+	1.05

a For these risk factor–disease pairs, RRs in the source were reported for all ages combined. We used median age at event and the age pattern of excess risk for serum total cholesterol and IHD to estimate RRs for each age category.

b The interventions studies replaced dietary SFA with PUFA, hence the RRs measure the effect of replacement. Effects of replacing PUFA for other macronutrients have not been evaluated in randomized interventions studies. However, evidence from cohort studies suggests that replacement of PUFA for carbohydrates, but not carbohydrates for SFA, would produce similar benefits [78], indicating that the measured benefits are due to PUFA.

**Table 3 pmed-1000058-t003:** Sources and magnitudes of relative risks (RRs) for the effects of categorical dietary risk factors on disease-specific mortality.

Risk Factor	Disease Outcome	Source of RR	Age Group	RR 1	RR 2	RR 3	RR 4	RR 5
**Low dietary omega-3 fatty acids** a	IHD^b^	Meta-analysis of randomized intervention studies and prospective cohort studies [31] c	30–44	2.18	1.80	1.46	1.14	1.00
			45–59	1.86	1.58	1.33	1.10	1.00
			60–69	1.41	1.28	1.16	1.05	1.00
			70–79	1.23	1.16	1.09	1.03	1.00
			80+	1.19	1.13	1.07	1.02	1.00
	Stroke^b^	Meta-analysis of 12 prospective cohort studies by authors^c^	30–44	1.27	1.19	1.11	1.04	1.00
			45–59	1.27	1.19	1.11	1.04	1.00
			60–69	1.16	1.11	1.06	1.02	1.00
			70–79	1.11	1.08	1.04	1.01	1.00
			80+	1.10	1.07	1.04	1.01	1.00

a Omega-3 intake categories in the analysis were (1) 0 to <62.5; (2) 62.5 to <125; (3) 125 to <187.5; (4) 187.5 to <250; and (5) ≥250 mg/d of eicosapentaenoic acid (EPA) and docosahexaenoic acid (DHA).

b For each disease outcome, RRs in the source were reported for all ages combined. We used median age at event and the age pattern of excess risk for serum total cholesterol and the same disease to estimate RRs for each age category.

c RRs were summarized via meta-regression across intake levels [79]. When RRs were reported for fish intake, we converted the units to omega 3 intake using the average omega-3 content of one serving of fish estimated using NHANES 2003–2004.

**Table 4 pmed-1000058-t004:** Sources and magnitudes of relative risks for the effects of alcohol use on disease-specific mortality.

Disease Outcome	Source of RR	Age Group	Sex	Abstainers	DI^a^	DII^a^	DIII^a^	Binge Drinkers
**IHD** a	Meta-analysis of observational studies for non-binge [19], [30] and binge drinkers [80]	30–44	—	1.00	0.60	0.62	1.00	1.00
		45–59	—	1.00	0.63	0.65	1.00	1.00
		60–69	—	1.00	0.82	0.83	1.00	1.00
		70–79	—	1.00	0.92	0.93	1.00	1.00
		80+	—	1.00	0.97	0.98	1.00	1.00
**Ischemic stroke** b	Meta-analysis of 35 observational studies [19], [81]	30–44	M	1.00	0.83	0.83	3.84	—
			F	1.00	0.88	1.07	1.33	—
		45–59	M	1.00	0.88	0.88	2.52	—
			F	1.00	0.91	1.05	1.22	—
		60–69	M	1.00	0.94	0.94	1.69	—
			F	1.00	0.96	1.02	1.10	—
		70–79	M	1.00	0.97	0.97	1.32	—
			F	1.00	0.98	1.01	1.05	—
		80+	M	1.00	1.00	1.00	1.00	—
			F	1.00	1.00	1.00	1.00	—
**Hemorrhagic stroke** b	Meta-analysis of 35 observational studies [19], [81]	30–44	M	1.00	1.65	3.16	6.65	—
			F	1.00	1.30	2.07	3.89	—
		45–59	M	1.00	1.42	2.21	3.60	—
			F	1.00	1.20	1.67	2.54	—
		60–69	M	1.00	1.19	1.55	2.18	—
			F	1.00	1.09	1.30	1.70	—
		70–79	M	1.00	1.09	1.25	1.55	—
			F	1.00	1.04	1.13	1.32	—
		80+	M	1.00	1.00	1.00	1.00	—
			F	1.00	1.00	1.00	1.00	—
**Hypertensive disease**	Overview of observational studies [19], [82], [83]	—	M	1.00	1.4	2.0	4.1	—
		—	F	1.00	1.4	2.0	2.0	—
**Cardiac arrhythmias**	Overview of observational studies [82]	—	—	1.00	1.51	2.23	2.23	—
**Breast cancer**	Systematic review of epidemiological studies [19], [82], [83]	30–44	F	1.00	1.15	1.41	1.46	—
		45+	F	1.00	1.14	1.38	1.62	—
**Colorectal cancer**	Pooled analysis of 8 prospective cohort studies [84]	—	M	1.00	1.0	1.16	1.41	—
		—	F	1.00	1.0	1.01	1.41	—
**Esophagus cancer**	Overview of observational studies [19], [82], [83]	—	—	1.00	1.80	2.38	4.36	—
**Mouth and pharynx cancer**	Overview of observational studies [19], [82], [83]	—	—	1.00	1.45	1.85	5.39	—
**Laryngeal cancer**	Overview of observational studies [82]	—	—	1.00	1.83	3.90	4.93	—
**Liver cancer**	Overview of observational studies [19], [82], [83]	—	—	1.00	1.45	3.03	3.60	—
**Selected other cancers** c	Overview of observational studies [19], [83]	—	—	1.00	1.1	1.3	1.7	—
**Diabetes mellitus**	Overview of observational studies [19], [82], [83]	—	M	1.00	0.99	0.57	0.73	—
		—	F	1.00	0.92	0.85	1.13	—
**Liver cirrhosis**	Overview of observational studies [19], [82], [83]	—	—	1.00	1.3	9.5	13	—
**Acute and chronic pancreatitis**	Meta-analysis of observational studies [85]	—	M	1.00	1.3	1.8	3.2	—
		—	F	1.00	1.3	1.8	1.8	—
								
			BAC %	<0.01	0.01–0.04	0.05–0.07	0.08–0.1	≥0.11
**Road traffic injury deaths**	Grand Rapids Study [19], [86] d		OR	1.0	1.2	1.7	4.0	10.7
**Falls, homicide and suicide, and other injury deaths**	Grand Rapids Study [19], [86] d		OR	10.7^e^	—	—	—	—

a Exposure categories were: Abstainer, a person not having had a drink containing alcohol within the last year; DI 0–19.99 g of pure alcohol daily (females) and 0–39.99 g (males); DII, 20–39.99 g (females) and 40–59.99 g (males); and DIII, >40 g (females) and >60 g (males). Binge drinking was defined as having at least one occasion of five or more drinks in the last month. For IHD, the categories refer to non-binge drinkers.

b For these risk factor–disease pairs, RRs in the source were reported for all ages combined. We used median age at event and the age pattern of excess risk from smoking and the same disease to estimate RRs for each age category.

c This category includes ICD-9 codes 210–239.

d These odds ratios were used to estimate PAF as described in the Methods section.

e Used to estimated PAF for having drunk alcohol in the last 6 h before injury.

**Table 5 pmed-1000058-t005:** Sources and magnitudes of relative risks for the effects of physical inactivity on disease-specific mortality.

Disease Outcome	Source of RR	Age Group	Highly Active	Recommended Level Active	Insufficiently Active	Inactive
**IHD**	Meta-analysis of 20 prospective cohort studies [87] a	30–69	1.00	1.15	1.66	1.97
		70–79	1.00	1.15	1.51	1.73
		80+	1.00	1.15	1.38	1.50
**Ischemic stroke**	Meta-analysis of 8 prospective cohort studies [87] a	30–69	1.00	1.12	1.23	1.72
		70–79	1.00	1.12	1.21	1.55
		80+	1.00	1.12	1.18	1.39
**Breast cancer**	Meta-analysis of 12 prospective cohort and 31 case-control studies [87] a	30–44	1.00	1.25	1.41	1.56
		45–69	1.00	1.25	1.41	1.67
		70–79	1.00	1.25	1.36	1.56
		80+	1.00	1.25	1.32	1.45
**Colon cancer**	Meta-analysis of 11 prospective cohort and 19 case-control studies [87] a	30–69	1.00	1.07	1.27	1.80
		70–79	1.00	1.07	1.21	1.59
		80+	1.00	1.07	1.16	1.39
**Diabetes**	Meta-analysis of 13 prospective cohort and 9 case-control studies [87] a	30–69	1.00	1.21	1.50	1.76
		70–79	1.00	1.21	1.43	1.60
		80+	1.00	1.21	1.34	1.45

Categories of physical activity were defined as below using responses to questions regarding physical activity during the past 30 d: inactive, no moderate or vigorous physical activity; low-active, <2.5 h/wk of moderate activity or <600 met·min/wk; moderately active: either ≥2.5 h/wk of moderate activity or ≥1 h of vigorous activity and ≥600 met·min/wk; highly active: ≥1 h/wk of vigorous activity and ≥1,600 met·min/wk.

a The meta-analysis of RRs for physical inactivity used three categories: inactive, insufficiently active, and recommended-level active. For this analysis, we re-scaled the RRs to set the highly active group as the reference category. The ratio of excess risk from recommended-level active to high-active was from Manson et al. for IHD [69], Hu et al. for ischemic stroke [70], Patel et al. 2003 for breast cancer [71], and Chao et al. for colon cancer [72].

**Table 6 pmed-1000058-t006:** Sources and magnitudes of relative risks for the effects of tobacco smoking on disease-specific mortality.

Disease Outcome	Source of RR	Age Group	Sex	RR
**IHD**	American Cancer Society Cancer Preventions Study, Phase II (ACS CPS-II) [88] a	30–44	M	5.51
			F	2.26
		45–59	M	3.04
			F	3.78
		60–69	M	1.88
			F	2.53
		70–79	M	1.44
			F	1.68
		80+	M	1.05
			F	1.38
**Stroke**	ACS CPS-II [88] a	30–44	M	3.12
			F	4.61
		45–59	M	3.12
			F	4.61
		60–69	M	1.88
			F	2.81
		70–79	M	1.39
			F	1.95
		80+	M	1.05
			F	1.00
**Hypertensive disease (sensitivity analysis)** b	ACS CPS-II [88] a	30–44	M	5.93
			F	2.38
		45–59	M	3.23
			F	4.05
		60–69	M	1.96
			F	2.67
		70–79	M	1.48
			F	1.74
		80+	M	1.06
			F	1.42
**Selected other cardiovascular diseases** b	ACS CPS-II [88] a	30–44	M	6.91
			F	2.65
		45–59	M	3.68
			F	4.65
		60–69	M	2.15
			F	3.00
		70–79	M	1.58
			F	1.89
		80+	M	1.07
			F	1.50
**Diabetes mellitus**	Meta-analysis of 25 prospective cohort studies with 1.2 million participants [89] a	—	—	1.44
**Lung cancer**	ACS CPS-II [90] a	—	M	21.3
			F	12.5
**Mouth, pharynx, and esophagus cancer**	ACS CPS-II [90] a	—	M	8.1
			F	6.0
**Stomach cancer**	ACS CPS-II [90] a	—	M	2.16
			F	1.49
**Liver cancer**	ACS CPS-II [90] a	—	M	2.33
			F	1.50
**Pancreas cancer**	ACS CPS-II [90] a	—	—	2.20
**Cervix uteri cancer**	ACS CPS-II [90] a	—	F	1.50
**Bladder cancer**	ACS CPS-II [90] a	—	M	3.00
			F	2.40
**Leukemia**	ACS CPS-II [90] a	—	M	1.89
			F	1.23
**Colorectal cancer (sensitivity analysis)**	ACS CPS-II [90], [91] a	—	M	1.32
			F	1.41
**Kidney and other urinary cancer**	ACS CPS-II [90] a	—	M	2.5
			F	1.5
**Chronic obstructive pulmonary disease**	ACS CPS-II [92] a	—	M	10.8
			F	12.3
**Other respiratory diseases** c	ACS CPS-II [92] a	—	M	1.90
			F	2.20
**Tuberculosis**	Meta-analysis of cohort, case-control, and cross-sectional studies [93]	—	—	1.62

a We used ACS CPS-II as the source of RRs because the Smoking Impact Ratio (SIR), which was used as the exposure metric for tobacco smoking in the main analysis, is calculated using ACS CPS-II cohort and because the study provided separate RRs for different cancers and cardiovascular diseases by age. The CPS-II RRs were also adjusted for multiple potential confounders.

b For these disease outcomes, RRs in the source were reported for all ages combined. We used median age at event and the age pattern of excess risk from IHD to estimate RRs for each age category.

c This category includes lower respiratory tract infections and asthma.

**Table 7:** Sources and magnitudes of relative risks for the effects of metabolic risk factors on disease-specific mortality.

Risk Factor	Disease Outcome	Source of RR	Units	Age Group	Sex	RR
**High blood glucose**	IHD	Meta-analysis of 19 prospective cohort studies with 237,000 participants [7] a	Per mmol/l increase	30–59	—	1.42
				60–69	—	1.20
				70+	—	1.20
	Stroke	Meta-analysis of 19 prospective cohort studies with 237,000 participants [7] a	Per mmol/l increase	30–59	—	1.36
				60–69	—	1.28
				70+	—	1.08
	Renal failure	Randomized trial of 3,900 participants [94]	Per mmol/l increase		—	1.26
**High LDL cholesterol**	IHD	Meta-analysis of ten prospective cohort studies [12]	Per mmol/l increase	30–44	—	2.94
				45–59	—	2.10
				60–69	—	1.59
				70–79	—	1.27
				80+	—	1.01
	Ischemic stroke b	Meta-analysis of nine prospective cohort studies [12]	Per mmol/l increase	30–44	—	1.30
				45–59	—	1.30
				60–69	—	1.18
				70–79	—	1.00^c^
				80+	—	1.00^c^
**High total cholesterol (sensitivity analysis)**	IHD	PSC meta-analysis of 61 prospective cohort studies with 900,000 European and North American participants [95]	Per mmol/l increase	30–44	—	2.11
				45–59	—	1.81
				60–69	—	1.39
				70–79	—	1.22
				80+	—	1.18
	Ischemic stroke	PSC [95]	Per mmol/l increase	30–44	—	1.51
				45–59	—	1.37
				60–69	—	1.12
				70–79	—	1.00^c^
				80+	—	1.00^c^
**High blood pressure**	IHD	PSC [11]	Per 20 mmHg increase	30–44	—	2.04
				45–59	—	2.01
				60–69	—	1.85
				70–79	—	1.67
				80+	—	1.49
	Stroke	PSC [11]	Per 20 mmHg increase	30–44	—	2.55
				45–59	—	2.74
				60–69	—	2.33
				70–79	—	2.00
				80+	—	1.49
	Hypertensive disease^b^	PSC [11]	Per 20 mmHg increase	30–44	—	4.78
				45–59	—	5.02
				60–69	—	4.55
				70–79	—	4.10
				80+	—	3.50
	Other cardiovascular diseases^d^	PSC [11]	Per 20 mmHg increase	30–44	—	2.52
				45–59	—	2.11
				60–69	—	1.89
				70–79	—	1.56
				80+	—	1.43
**Overweight–obesity (high BMI)**	IHD	APCSC meta-analysis of 33 prospective cohorts with 310,000 participants [65] e,f	Per kg/m^2^ increase	30–44	—	1.14
				45–59	—	1.09
				60–69	—	1.08
				70–79	—	1.05
				80+	—	1.02
	Ischemic stroke	APCSC [65]	Per kg/m^2^ increase	30–44	—	1.14
				45–59	—	1.10
				60–69	—	1.08
				70–79	—	1.05
				80+	—	1.03
	Hypertensive disease	APCSC [65]	Per kg/m^2^ increase	30–44	—	1.22
				45–59	—	1.18
				60–69	—	1.14
				70–79	—	1.11
				80+	—	1.08
	Postmenopausal breast cancer	Meta-analysis of 31 prospective cohort studies [17]	Per kg/m^2^ increase	45+	F	1.02
	Colon cancer	Meta-analysis of 22 prospective cohort studies in males and 19 in females [17]	Per kg/m^2^ increase	—	M	1.04
					F	1.02
	Corpus uteri cancer	Meta-analysis of 19 prospective cohort studies [17]	Per kg/m^2^ increase	—	F	1.10
	Kidney cancer	Meta-analysis of 11 prospective cohort studies in males and 12 in females [17]	Per kg/m^2^ increase	—		1.05
	Pancreatic cancer	Meta-analysis of 12 prospective cohort studies in males and 11 in females [17]	Per kg/m^2^ increase	—	M	1.01
					F	1.02
	Non-Hodgkin lymphoma (sensitivity analysis)	Meta-analysis of six prospective cohort studies in males and seven in females [17]	Per kg/m^2^ increase	—	—	1.01
	Diabetes mellitus	APCSC meta-analysis prospective cohort studies with 150,000 participants [96]	Per kg/m^2^ increase	30–59	—	1.20
				60–69	—	1.16
				70+	—	1.11

a See Danaei et al. [61] for sensitivity to using RRs from systematic reviews of other epidemiological studies.

b For these risk factor–disease pairs, RRs in the source were reported for all ages combined. We used median age at event and the age pattern of excess risk from another risk factor and the same disease (e.g., age pattern of total serum cholesterol and ischemic stroke was applied to LDL and ischemic stroke) or from the same risk factor and another disease (e.g., age pattern of excess risk for SBP and all cardiovascular diseases was applied to SBP and hypertensive disease) to estimate RRs for each age category.

c We used a null association in those 70-y-old and older because RRs in two large meta-analyses of prospective studies [95], [97] were not statistically significant from null, and did not show consistent benefits for lower total cholesterol in these ages. There is some evidence from clinical trials that statins reduce the risk of stroke in older ages [98]. However, statins may reduce stroke mortality through other, non-cholesterol mechanisms such as stabilization of atherosclerotic plaques [99]. In the sensitivity analysis for high LDL cholesterol and ischemic stroke, we used an RR of 1.12 in these age groups.

d This category includes rheumatic heart disease, acute and subacute endocarditis, cardiomyopathy, other inflammatory cardiac diseases, valvular disorders, aortic aneurysm, pulmonary embolism, conduction disorders, peripheral vascular disorders, and other ill-defined cardiovascular diseases.

e We used meta-analyses of studies with measured weight and height because using self-reported weight and height can lead to bias in estimated RRs. The correlation between self-reported and measured weight, as found in selected studies [100], [101], does not remove the possibility of bias because even with perfect correlation, the absolute bias in self-reported weight and height may be a function of its true value.

f The RRs reported for Asian and Australia–New Zealand populations were not significantly different in this meta-analysis providing empirical evidence on absence of significant effect modification in the multiplicative scale by ethnicity. A meta-analysis of studies in Europe and North America included studies [102] with self-reported height and weight and was thus not used in this analysis. The RRs reported in that meta-analysis ranged from 1.02 to 1.26 and the average RR weighted by number of cases was 1.07 per kg/m^2^ which is almost equal to the RR for 60- to 69-y-olds in this analysis.

APCSC, Asia-Pacific Cohorts Studies Collaboration; PSC, Prospective Studies Collaboration.

The studies used for etiological effect sizes included both randomized intervention studies of exposure reduction and observational studies (primarily prospective cohort studies) that estimated the effects of baseline exposure. The majority of observational studies used for effect sizes had adjusted for important potential confounding factors. Each RR used in our analysis represents the best evidence for the proportional effect of risk factor exposure on disease-specific mortality in the population based on the current causes and determinants of the population distribution of exposure (see also Discussion).

We used RRs for blood pressure, LDL cholesterol, and FPG that were adjusted for regression dilution bias using studies that had repeated exposure measurement [7],[11], [12]; for blood pressure and LDL cholesterol, the adjusted magnitude is supported by effect sizes from randomized studies [13],[14]. Evidence from a large prospective study with multiple measurements of weight and height showed that regression dilution bias did not affect the RRs for BMI, possibly because there is less variability [15]. RRs for dietary salt and PUFA-SFA replacement were from intervention studies, and hence unlikely to be affected by regression dilution bias. RRs for dietary trans fatty acids were primarily from studies that had used cumulative averaging of repeated measurements [16] that reduces but may not fully correct for regression dilution bias. RRs for physical inactivity, alcohol use, smoking, and dietary omega-3 fatty acids and fruits and vegetables were not corrected for regression dilution bias due to insufficient current information from epidemiological studies on exposure measurement error and variability, which is especially important when error and variability of self-reported exposure may themselves differ across studies.

For each risk factor–disease pair, we used the same RR for men and women except where empirical evidence indicated that the RR differed by sex: colon and pancreas cancers caused by high BMI [17], and all disease outcomes caused by alcohol use and tobacco smoking, for which there are sex differences in factors such as smoking duration and intensity [18] and type of alcohol consumed [19]. The RRs for some risk factor–disease associations vary by age, especially for cardiovascular diseases. We used consistent age-varying distributions of RRs across risk factors and diseases (Tables 2–7).

The current evidence suggests that when measured comparably the proportional effects of the risk factors considered in this analysis are similar across populations, e.g., Western and Asian populations [7],[20],[21]. The exception to this observation is the effects of alcohol use on ischemic heart disease (IHD) where the pattern of drinking (regular versus binge) determines the RR. We used both the average quantity of alcohol consumed as well as the drinking pattern in our analysis of exposure and RRs for alcohol use and IHD. The effects of alcohol on injuries and violence may also be modified by social, policy, and transportation factors. Therefore, we did not pool epidemiological studies on the injury effects of alcohol from different countries, but used data sources that appropriately measure effects in the US (Table 4).

#### Disease-specific deaths

The number of disease-specific deaths, by age and sex, was obtained from the NCHS, which maintains records for all deaths in the US. Although the US has automated (computerized) assignment of an International Classification of Diseases (ICD) code for the underlying cause of death, the validity and comparability of cause of death statistics may be affected at the time of medical certification, especially for cardiovascular causes and diabetes [22]–[24]. We adjusted for incomparability in cause of death assignment using previously described methods [22],[23]. This adjustment required information on multiple contributing causes of death and county of residence. We obtained county identifiers for all deaths in 2005 through a special request to the NCHS.

Several risk factors have different effects on ischemic and hemorrhagic stroke (Table 1). Slightly more than 50% of stroke deaths in 2005 were assigned to unspecified subtype (ICD-10 code I-64). We redistributed these deaths to ischemic and hemorrhagic stroke using proportions from large epidemiological studies with high-quality diagnosis and cause-of-death assignment [25], stratified by age using a meta-analysis of stroke registries in Western populations [26].

### Estimating Mortality Attributable to Risk Factors

For each risk factor and for each disease causally associated with its exposure, we computed the proportional reduction in disease-specific deaths that would occur if risk factor exposure had been reduced to an alternative level. This is known as the population-attributable fraction (PAF) and measures the total effects of a risk factor (direct as well as mediated through other factors). For risks measured continuously (blood pressure, BMI, LDL cholesterol, FPG, dietary fruits and vegetables, and trans and polyunsaturated fatty acids), we computed PAFs using the following relationship.
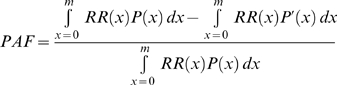
(1)Where *x* = exposure level; *P*(*x*) = actual distribution of exposure in the population; *P*′(*x*) = alternative distribution of exposure in the population; *RR*(*x*) = relative risk of mortality at exposure level *x*; and *m* = maximum exposure level.

For risks measured in categories of exposure (smoking, physical inactivity, alcohol use, and dietary omega-3 fatty acids), we used the discrete version of the same estimator for PAF.

We used a different method of estimating the PAFs for effects of alcohol use on injuries. A number of emergency room studies have collected information on alcohol consumption in the 6 h prior to the injury among injury patients. Injuries that occur among patients who had consumed alcohol prior to their injury were classified as “alcohol-related” injuries. Because some of these injuries would have occurred in the absence of alcohol, not all are caused by alcohol use; in other words, the proportion of alcohol-attributable injuries is lower than that of alcohol-related injuries. Highway studies have quantified the increased risk of road traffic deaths among drivers who have consumed alcohol according to the drivers' blood alcohol concentration, often reported as odds ratios (ORs). Ideally, ORs would be used in conjunction with data on population prevalence of intoxication to calculate PAF. Because intoxication data were not available, we used a slightly modified equation to calculate the PAF using ORs from highway studies and data on alcohol-related injuries:
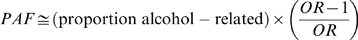
(2)


The proportion of alcohol-related injuries was obtained from Fatality Analysis Reporting System (FARS) for road traffic injuries and from a meta-analysis of emergency room studies for other types of intentional and unintentional injuries [27], [28]. FARS is a census of fatal crashes maintained by the National Highway Traffic Safety Administration and includes information on the blood alcohol concentration (BAC) level of drivers involved in fatal crashes, regardless of whether the decedent was the driver or not. Beginning in 2001, National Center for Statistics and Analysis uses a multiple imputation method to impute ten values for each missing BAC value. Additional information on FARS is available at http://www-fars.nhtsa.dot.gov/Main/index.aspx. The sources for ORs are provided in Table 4.

We calculated the number of deaths from each causally related disease outcome attributable to a risk factor by multiplying its PAF by total deaths from that disease. Disease-specific deaths attributable to each risk factor were summed to obtain the total (all-cause) attributable deaths. Deaths from different diseases attributable to a single risk factor are additive because in mortality statistics based on the ICD, each death is categorically assigned to a single underlying cause (disease) with no overlap between disease-specific deaths. However, the deaths attributable to individual risk factors often overlap and should not be summed (see Discussion).

To measure the mortality effects of all non-optimal levels of exposure consistently and comparably across risk factors, we used an optimal exposure distribution, referred to as the theoretical-minimum-risk exposure distribution (TMRED), as the alternative exposure distribution (Table 1). The TMREDs were zero for risk factors for which zero exposure led to minimum risk (e.g., no tobacco smoking). For BMI, blood pressure, blood glucose, and LDL cholesterol, zero exposure is physiologically impossible. For these risks we used TMREDs based on the levels corresponding to the lowest mortality rate in epidemiological studies or the levels observed in low-exposure populations (Table 1). Alcohol use may be beneficial or harmful depending on the specific disease outcome and patterns of alcohol consumption [29], [30]. We used a TMRED of zero for alcohol in our primary analysis, and regular drinking of small amounts as the TMRED in a sensitivity analysis. The TMREDs for factors with protective effects (physical activity and dietary PUFA-SFA replacement, omega-3 fatty acids, and fruits and vegetables) were selected as the intake and activity levels to which beneficial effects may plausibly continue based on the evidence from current studies. For example, intake of omega-3 fatty acids seems to reduce IHD mortality at intakes up to 250 mg/d, but has relatively little additional mortality benefits at higher intakes [31]. In setting TMREDs for protective factors, we also took into account the levels observed in populations that have high intake, e.g., for fruits and vegetables.

We conducted all analyses separately by sex and age group (30–44, 45–59, 60–69, 70–79, and ≥80 y). We restricted analyses to ≥30 y because there are limited data on the mortality effects of these risk factors at younger ages and because there are few deaths from diseases affected by these risks in younger ages (about 10,000 deaths from the relevant non-injury causes in Americans <30 y versus 1,745,000 in those ≥30 y). The exception was the effect of alcohol use on injuries for which we also included 0- to 29-y-olds because there are substantial injury deaths at these ages. Therefore, we can assess both the role of alcohol use as a cause of injuries in young drinkers and the effect of alcohol use by any drinker (e.g., an intoxicated driver) on injury in young nondrinkers.

### Uncertainty and Sensitivity Analyses

We estimated the uncertainty of the number of deaths attributable to each risk factor as caused by sampling variability. To compute sampling uncertainty, we used a simulation approach to combine the uncertainties of exposure distributions and RRs in each age–sex group. In the simulation method, we drew repeatedly from the distributions of exposure mean and SD (for continuous risks) or prevalence in each exposure category (for categorical risks). The uncertainty of these parameters was characterized using normal, Chi-square, or binomial distributions. RRs for each disease were drawn from a log-normal distribution independently from exposure. Each set of exposure and disease-specific RR draws was used to calculate the PAFs for all diseases associated with the risk factor, separately by age and sex. We used 500 draws for each risk factor, and report 95% confidence intervals (CIs) based on the resulting distributions of 500 estimated attributable deaths. Further simulation details and computer code are available from the authors by request.

In addition to sampling uncertainty, we examined the sensitivity of our results to important methodological factors and data sources. The methodological factors and data sources in the sensitivity analyses included the choice of exposure metrics, the shape of the exposure distribution, the TMREDs, disease outcomes causally associated with risk factors, and etiological effect sizes (Table S1).

We used RRs adjusted for major potential confounders to estimate the causal components of risk factor–disease associations. However, if there is also a correlation between exposure and disease-specific mortality, due to correlations of exposure with other risks or other unobserved factors, the above equations may result in under- (when there is positive correlation) or over-estimation (negative correlation) of the true PAF when used with adjusted RRs [32]–[36]. To assess the effect of correlation, we also calculated PAFs that incorporated correlations between risk factors or between risk factors and underlying disease-specific mortality in multiple sensitivity analyses. Ideally the analyses of risk factor correlations would have used the complete multivariate distribution of exposure to all risk factors and disease outcomes. However, the sources in this analysis did not provide data on the joint exposure distributions of all risk factors together. Therefore, our analyses of risk factor correlation using current data sources were limited to risk factor pairs.

Analyses were conducted using Stata version 10 (Stata Corp, College Station, Texas) and SAS version 9.1 (SAS Institute, Cary, NC).

## Results

In the year 2005, 2,448,017 US residents died; 49% of these deaths were among men. Ninety-six percent of all deaths in the US were in people ≥30 y of age. After adjustment for comparability of cause-of-death assignment [22],[23], the four most common causes of death were IHD (434,000 deaths), lung cancer (163,000 deaths), stroke (150,000 deaths), and chronic obstructive pulmonary diseases (124,000 deaths).

### Total Mortality Effect of Risk Factors

**Figure 1:**
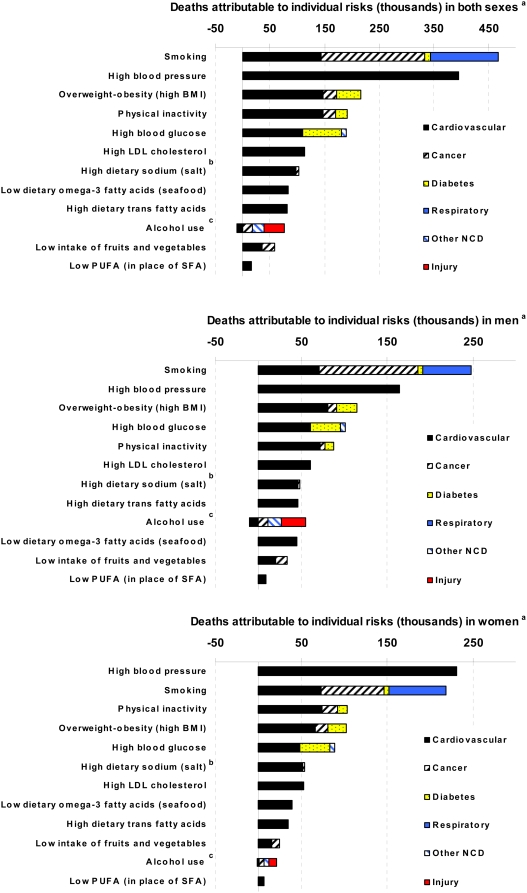
Deaths attributable to total effects of individual risk factors, by disease. Data are shown for both sexes combined (upper graph); men (middle graph); and women (lower graph). See Table 8 for 95% CIs. Notes: We used RRs for blood pressure, LDL cholesterol, and FPG that were adjusted for regression dilution bias using studies that had repeated exposure measurement [7],[11],[12]; for blood pressure and LDL cholesterol, the adjusted magnitude is supported by effect sizes from randomized studies [13],[14]. Evidence from a large prospective study using multiple measurements of weight and height showed that regression dilution bias did not affect the RRs for BMI, possibly because there is less variability [15]. RRs for dietary salt and PUFA were from intervention studies, and hence unlikely to be affected by regression dilution bias. RRs for dietary trans fatty acids were primarily from studies that had used cumulative averaging of repeated measurements [16] that reduces but may not fully correct for regression dilution bias. RRs for physical inactivity, alcohol use, smoking, and dietary omega-3 fatty acids and fruits and vegetables were not corrected for regression dilution bias due to insufficient current information from epidemiological studies on exposure measurement error and variability, which is especially important when error and variability of self-reported exposure may themselves differ across studies. Regression dilution bias often, although not always, underestimates RRs in multivariate analysis [48]. ^a^The figures show deaths attributable to the total effects of each individual risk. There is overlap between the effects of risk factors because of multicausality and because the effects of some risk factors are partly mediated through other risks. Therefore, the number of deaths attributable to individual risks cannot be added. ^b^The effect of high dietary salt on cardiovascular diseases was estimated through its measured effects on systolic blood pressure. ^c^The protective effects of alcohol use on cardiovascular diseases are its net effects. Regular moderate alcohol use is protective for IHD, ischemic stroke, and diabetes, but any use is hazardous for hypertensive disease, hemorrhagic stroke, cardiac arrhythmias, and other cardiovascular diseases. NCD, noncommunicable diseases.

Tobacco smoking was responsible for an estimated 467,000 (95% CI 436,000–500,000) deaths and high blood pressure for 395,000 (372,000–414,000) deaths, each accounting for about one in five or six deaths in US adults in 2005 (Figure 1A, Table 8). Overweight–obesity, physical inactivity, and high blood glucose each caused 190,000–216,000 deaths (8%–9% of all deaths in adults). The mortality effects of individual dietary risk factors ranged from 15,000 deaths for low dietary PUFA (<1% of all deaths) to 82,000–102,000 deaths for low dietary omega-3 fatty acids, high dietary trans fatty acids, and high dietary salt. Alcohol use caused 90,000 deaths from road traffic and other injuries, violence, chronic liver disease, cancers, alcohol use disorders, hemorrhagic stroke, arrhythmias and hypertensive disease, but also averted a balance of 26,000 deaths from IHD, ischemic stroke, and diabetes, due to benefits among those who drank alcohol moderately and regularly.

**Table 8:** Deaths from all causes (thousands of deaths) attributable to risk factors and the 95% confidence intervals of their sampling uncertainty.

Risk factor	Male	Female	Both Sexes
Tobacco smoking	248 (226–269)	219 (196–244)	467 (436–500)
High blood pressure	164 (153–175)	231 (213–249)	395 (372–414)
Overweight–obesity (high BMI)	114 (95–128)	102 (80–119)	216 (188–237)
Physical inactivity	88 (72–105)	103 (80–128)	191 (164–222)
High blood glucose	102 (80–122)	89 (69–108)	190 (163–217)
High LDL cholesterol	60 (42–70)	53 (44–59)	113 (94–124)
High dietary salt (sodium)	49 (46–51)	54 (50–57)	102 (97–107)
Low dietary omega-3 fatty acids (seafood)	45 (37–52)	39 (31–47)	84 (72–96)
High dietary trans fatty acids	46 (33–58)	35 (23–46)	82 (63–97)
Alcohol use^a^	45 (32–49)	20 (17–22)	64 (51–69)
Low intake of fruits and vegetables	33 (23–45)	24 (15–36)	58 (44–74)
Low dietary polyunsaturated fatty acids (PUFA) (in replacement of SFA)	9 (6–12)	6 (3–9)	15 (11–20)

a Excludes uncertainty in intentional and unintentional injury outcomes because the attributable deaths used data sources that did not report sampling uncertainty.

### Mortality Effects of Risk Factors by Disease

Most deaths attributable to these risks were from cardiovascular diseases (Figure 1). Cancers, respiratory diseases, diabetes, and injuries nonetheless accounted for at least 23% of all deaths caused by smoking, alcohol use, high blood glucose, physical inactivity, low intake of fruits and vegetables, and overweight–obesity. The single largest risk factor for cardiovascular mortality in the US was high blood pressure, responsible for an estimated 395,000 (95% CI 372,000–414,000) cardiovascular deaths (45% of all cardiovascular deaths), followed by overweight–obesity, physical inactivity, high LDL cholesterol, smoking, high dietary salt, high dietary trans fatty acids, and low dietary omega-3 fatty acids. Smoking had the largest effect on cancer mortality compared with any other risk factor, causing an estimated 190,000 (184,000–194,000) or 33% of all cancer deaths.

### Mortality Effects of Risk Factors by Sex and Age

High blood pressure was the leading cause of death in women (231,000 deaths [95% CI 213,000–249,000], 19% of all female deaths), whereas smoking remains the leading cause of death in men (248,000 deaths [226,000–269,000], 21% of all male deaths). The leading causes of death in men and women were different because women have higher blood pressure and men higher cumulative (i.e., current and former) smoking. Overweight–obesity, physical inactivity, and high blood glucose were the third to fifth causes of death for both sexes (Figure 1B and 1C). High dietary salt was responsible for slightly more deaths than high LDL cholesterol in women.

The mortality effects of all individual risk factors except alcohol use were almost equally divided between men and women (i.e., at least 40% of deaths attributable to each individual risk factor were either in men or in women). Seventy percent of all deaths attributable to alcohol use occurred in men (45,000 deaths), because men consumed more alcohol and had more binge drinking.

**Figure 2:**
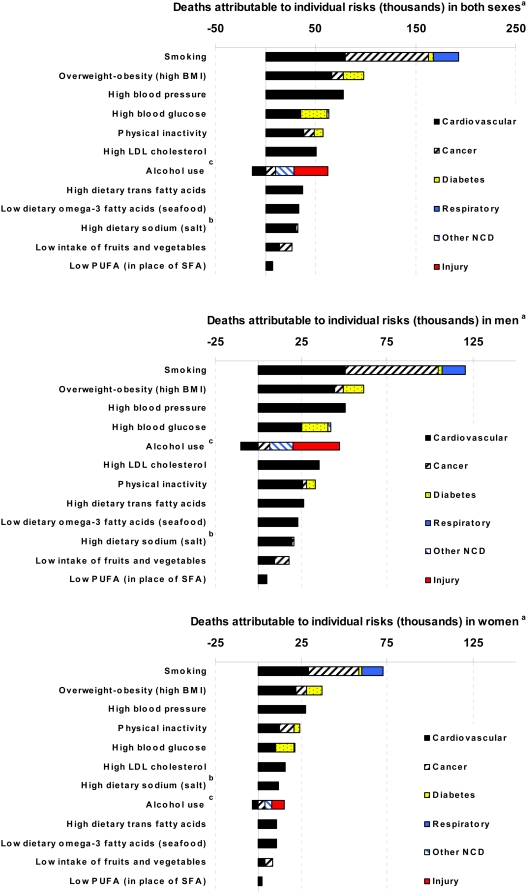
Deaths attributable to total effects of individual risk factors, by disease in those below 70 years of age. Data are shown for both sexes combined (upper graph); men (middle graph); and women (lower graph). See Figure 1 notes.

Four percent of all deaths in the US occurred in people between 30 and 45 y of age. No individual risk factor was responsible for more than 7% of deaths in this age group. However, this age group bore 34% of alcohol-caused injuries (Table 9), making injury deaths in young adults the major mortality impact of alcohol use. Eighty percent of deaths attributable to high blood pressure and 68% and 70% of those attributable to high dietary salt and physical inactivity, respectively, occurred after 70 y of age (Table 9). Conversely, 40% or more of all deaths attributable to high LDL cholesterol, overweight–obesity, high dietary trans fatty acids, low dietary PUFA and omega-3 fatty acids, low intake of fruits and vegetables, alcohol use, and smoking occurred before 70 y of age (Table 9). As a result, when the young and middle-aged (≤70 y of age) mortality effects of these risk factors were evaluated, smoking was by far the leading cause of death in both men and women ≤70 y, followed by overweight–obesity (Figure 2).

**Table 9:** Distribution of cause-specific and all-cause deaths attributable to risk factors by age group and by sex.

Risk Factor	Disease	0–29 y	30–45 y	45–69 y	≥ 70 y	Males	Females
**High blood glucose**	Cardiovascular diseases	NA	2 (1 to 3)	31 (24 to 40)	68 (58 to 75)	55 (43 to 68)	45 (32 to 57)
	Diabetes mellitus^a^	NA	3 (3 to 3)	33 (33 to 33)	64 (64 to 64)	51 (51 to 51)	49 (49 to 49)
	Renal failure	NA	1 (0 to 6)	21 (3 to 71)	77 (26 to 96)	53 (12 to 94)	47 (6 to 88)
	All causes	NA	2 (2 to 3)	31 (26 to 36)	67 (61 to 72)	53 (46 to 61)	47 (39 to 54)
**High LDL cholesterol**	Cardiovascular diseases	NA	4 (0 to 6)	40 (30 to 47)	55 (50 to 66)	53 (44 to 59)	47 (41 to 56)
**High blood pressure**	Cardiovascular diseases	NA	1 (1 to 1)	19 (18 to 20)	80 (79 to 82)	42 (39 to 44)	58 (56 to 61)
**Overweight–obesity (high BMI)**	Cardiovascular diseases	NA	5 (3 to 6)	41 (33 to 48)	55 (47 to 63)	55 (47 to 65)	45 (35 to 53)
	Cancers	NA	2 (2 to 3)	42 (38 to 47)	55 (51 to 60)	40 (36 to 46)	60 (54 to 64)
	Diabetes mellitus	NA	5 (4 to 5)	42 (38 to 47)	54 (48 to 58)	52 (46 to 58)	48 (42 to 54)
	All causes	NA	4 (3 to 5)	41 (36 to 46)	55 (49 to 61)	53 (47 to 60)	47 (40 to 53)
**High dietary trans fatty acids**	Cardiovascular diseases	NA	5 (3 to 7)	41 (31 to 50)	54 (45 to 65)	57 (46 to 67)	43 (33 to 54)
**Low dietary polyunsaturated fatty acids (PUFA) (in replacement of SFA)**	Cardiovascular diseases	NA	7 (2 to 11)	40 (23 to 56)	53 (37 to 70)	59 (43 to 75)	41 (25 to 57)
**Low dietary omega-3 fatty acids**	Cardiovascular diseases	NA	4 (3, 5)	36 (30 to 41)	60 (54 to 66)	53 (47 to 60)	47 (40 to 53)
**High dietary salt**	Cardiovascular diseases	NA	3 (3 to 3)	28 (27 to 30)	69 (67 to 70)	47 (45 to 50)	53 (50 to 55)
	Cancers	NA	5 (1 to 8)	36 (21 to 52)	59 (43 to 74)	58 (40 to 73)	42 (27 to 60)
	All causes	NA	3 (3 to 3)	29 (27 to 30)	68 (66 to 70)	48 (45 to 50)	52 (50 to 55)
**Low intake of fruits and vegetables**	Cardiovascular diseases	NA	3 (1 to 5)	35 (22 to 52)	62 (44 to 75)	55 (37 to 76)	45 (24 to 63)
	Cancers	NA	3 (2 to 5)	56 (39 to 71)	41 (25 to 58)	62 (47 to 76)	38 (24 to 53)
	All causes	NA	3 (2 to 5)	43 (32 to 57)	54 (39 to 66)	58 (45 to 71)	42 (29 to 55)
**Alcohol use** b	Cardiovascular diseases	NA	11 (4 to 34)	131 (93 to 159)	−42 (−75 to −7)	105 (85 to 126)	−5 (−26 to 15)
	Cancers	NA	5 (4 to 6)	55 (49 to 61)	40 (34 to 46)	64 (58 to 69)	36 (31 to 42)
	Diabetes mellitus	NA	5 (4 to 6)	44 (40 to 49)	51 (46 to 55)	50 (45 to 55)	50 (45 to 55)
	Other noncommunicable diseases^c^	NA	15 (14 to 16)	68 (66 to 71)	17 (15 to 19)	74 (72 to 76)	26 (24 to 28)
	Injuries^d^	31 (31 to 31)	34 (34 to 34)	29 (29 to 29)	6 (6 to 6)	77 (77 to 77)	23 (23 to 23)
	All causes	18 (16 to 23)	24 (21 to 30)	34 (20 to 40)	24 (20 to 30)	70 (62 to 73)	30 (27 to 38)
**Physical inactivity**	Cardiovascular diseases	NA	2 (1 to 2)	24 (19 to 30)	74 (68 to 79)	49 (40 to 60)	51 (40 to 60)
	Cancers	NA	5 (3 to 7)	42 (35 to 50)	53 (45 to 60)	24 (18 to 29)	76 (71 to 82)
	Diabetes mellitus	NA	3 (2 to 5)	35 (28 to 43)	61 (52 to 69)	50 (40 to 61)	50 (39 to 60)
	All causes	NA	2 (2 to 3)	28 (23 to 33)	70 (64 to 75)	46 (38 to 54)	54 (46 to 62)
**Tobacco smoking**	Cardiovascular diseases	NA	4 (0 to 7)	51 (43 to 63)	44 (34 to 54)	49 (38 to 60)	51 (40 to 62)
	Cancers	NA	1 (0 to 2)	43 (42 to 44)	56 (55 to 57)	61 (60 to 62)	39 (38 to 40)
	Other respiratory diseases^e^	NA	0 (0 to 1)	21 (19 to 22)	79 (78 to 80)	46 (44 to 48)	54 (52 to 56)
	Diabetes mellitus	NA	1 (0 to 3)	36 (30 to 41)	63 (57 to 68)	50 (44 to 57)	50 (43 to 56)
	All causes	NA	2 (0 to 3)	39 (36 to 42)	59 (56 to 62)	53 (49 to 57)	47 (43 to 51)

Numbers show percent in each age group or in each sex and the corresponding 95% confidence intervals of sampling uncertainty.

a There is no sampling uncertainty for this outcome because all the deaths due to diabetes are by definition attributable to high blood glucose.

b The negative proportions for alcohol use and cardiovascular diseases in older ages and in females occur because the protective effects are larger than the hazardous effects.

c This category includes liver cirrhosis, acute and chronic pancreatitis, and alcohol use disorders.

d We did not estimate sampling uncertainty for injury outcomes because the attributable deaths used data sources that did not report sampling uncertainty.

e This category includes lower respiratory tract infections, asthma, and tuberculosis.

### Mortality Effects of Risk Factor by Exposure Level

There was substantial variation in how deaths attributable to these risks were distributed below or above commonly used thresholds and guidelines (Table 10): close to two-thirds of deaths attributable to high blood pressure (66%), high BMI (63%), and high blood glucose (60%) occurred in people who would be clinically classified as hypertensive, obese, or diabetic, even though these groups make up only 10%–33% of the US adult population (note that the estimated benefits in these people would be achieved if risk factor levels are reduced to their TMREDs, and not simply to the clinical threshold). In contrast, more than one-half of deaths attributable to high LDL cholesterol were among people below the conventional threshold for defining dyslipidemia (3.37 mmol/l).

**Table 10:** Distribution of risk factor exposure and attributable deaths by ranges or categories of exposure defined using common clinical and public health thresholds and guidelines.

Risk Factor	Source of Definition for Categories	Exposure Categories	Percentage of Attributable Deaths	Percentage of Population (≥30 Years Old)
**High blood glucose** a	Definition of diabetes (FPG≥7 mmol/l) and impaired FPG (FPG 5.56 to 6.99 mmol/l) by American Diabetes Association [103]	FPG≥7 mmol/l	60	10
		FPG 5.56–6.99 mmol/l	34	29
		FPG<5.56 mmol/l	6	61
**High LDL cholesterol**	Definition of high LDL cholesterol in low risk (4.14 mmol/l) and moderate risk (3.37 mmol/l) individuals in Adult Treatment Panel III guidelines [104]	LDL≥4.14 mmol/l	5	11
		LDL 3.37–4.13 mmol/l	30	22
		LDL<3.37 mmol/l	65	67
**High blood pressure**	Definition of hypertension (SBP≥140 mmHg) [105]	SBP≥140 mmHg	66	15
		SBP<140 mmHg	34	85
**Overweight–obesity (high BMI)**	Definition of obesity (BMI≥30 kg/m^2^) and overweight (BMI 25 to 29.9 kg/m^2^)	BMI≥30 kg/m^2^	63	33
		BMI 25–29.9 kg/m^2^	29	33
		BMI<25 kg/m^2^	8	33
**High dietary salt**	Recommended level of dietary sodium (<100 mmol/d) by American Heart Association [106]	Dietary sodium≥100 mmol/d	88	75
		Dietary sodium<100 mmol/d	12	25
**Physical inactivity**	Definition of moderately active (600 met·min/wk) is the same as the recommended level of activity by Centers for Disease Control and Prevention [107]	Inactive	74	31
		Low-active	19	25
		Moderately active	7	23
		Highly active	0	21
**Tobacco smoking**	—	Current smokers	43	25
		Former smokers	57	25
		Never smokers	0	50

The proportion of population and mortality effects in different exposure categories. We have not included dietary risks other than dietary salt in this table primarily because current guidelines do not recommend a specific level of intake.

a Deaths assigned to diabetes mellitus in the vital statistics and deaths attributable to renal failure are included in the ≥7 mmol/l category because all individuals whose deaths are assigned to diabetes or diabetic renal failure would, by definition, have been diagnosed with diabetes disease, and hence have FPG ≥7 mmol/l.

The burden of smoking was almost equally distributed among current and former smokers, because harmful effects continue among many Americans who have quit smoking. Twenty-nine percent of the chronic disease mortality effects of alcohol use occurred among heavy drinkers (i.e., men who consumed more than 60 grams of pure alcohol or 4 drinks per day and women who consumed more than 40 grams per day); this group did not have any mortality benefits from alcohol use. In contrast, in those who had light alcohol consumption (up to 40 g per day for men and 20 g per day for women), the protective effects on IHD and diabetes mortality were larger than the hazardous effects from other chronic diseases, leading to an overall reduction in mortality in this group (unpublished results).

### Sensitivity Analyses

The results of the sensitivity analyses in Table S1 show that the estimated numbers of deaths attributable to risk factors were most sensitive to the choice of the optimal exposure distribution (the TMRED) to which current risk factor exposure distributions were compared. For example, if the TMREDs for LDL cholesterol and BMI were 2.3 (instead of 2.0) mmol/l and 23 (instead of 21) kg/m^2^, respectively, the number of deaths attributable to them would be 18% and 19% lower. Similarly, lowering the TMRED of physical activity to the (less ambitious) current recommended level of 600 met·min per week (equivalent to 20 min of moderate activity every day) would prevent 62,000 (32%) fewer deaths than if people pursued a higher goal of 1,600 met·min per week (including at least one hour of vigorous activity per day). The TMRED for alcohol use must balance its harmful and beneficial effects. If the entire adult US population had light alcohol consumption, a total of 12,000 cardiovascular deaths would be prevented, largely among adults aged ≥45 y. However, this level of alcohol consumption would also cause an estimated 8,000 deaths due to road traffic accidents largely among adults aged <30 y.

Incorporating correlation of a risk factor with disease-specific mortality and with other risks changed the estimated number of deaths attributable to a risk factor by 3%–31%, depending on the specific risk factor and disease. The results were robust to whether exposure in the population was approximated with a normal distribution and to the inclusion of the few disease outcomes for which the evidence of causal association was weaker. Mortality effects of dietary salt were sensitive to the magnitude of its effects on SBP, because there was an almost 2-fold difference between two separate meta-analyses of salt reduction trials [37],[38].

## Discussion

Our analysis of the mortality effects of major dietary, lifestyle, and metabolic risk factors in the US using comparable methods showed that tobacco smoking and high blood pressure were the leading risk factors for mortality, responsible for nearly one in five and one in six deaths in US adults, respectively. The large effects of tobacco smoking were caused by long-term cumulative exposure in current smokers as well as the remaining effects in former smokers, especially in men. The large numbers of deaths attributable to high blood pressure were related to high exposure levels, particularly in women [39]. Overweight–obesity, physical inactivity, and high blood glucose each caused about one in ten deaths, and both affected women disproportionately more than men. In those younger than 70 y of age, tobacco smoking was by far the leading modifiable cause of death, and overweight–obesity caused more deaths than did high blood pressure. Other lifestyle, metabolic, and dietary risk factors for chronic diseases also caused significant adult mortality, although their individual effects were 3%–24% of those of smoking. A comparison of our results with those of other risk factors is shown in Table S2. This comparison was done only for those risk factors included in previous analyses, because these analyses had included substantially fewer metabolic and dietary risks than ours.

Each RR used in our analysis represents the best evidence for the impact of risk factor exposure on disease-specific mortality in the population, based on the current causes and determinants of the population distribution of exposure. The mortality effects of a risk factor may depend on whether an expected increase in exposure is prevented or whether exposure is reduced after it has risen. It may also depend on the specific intervention used to prevent or reverse risk factor exposure. The estimated effects of blood pressure, LDL cholesterol, omega-3 fatty acids, and PUFA-SFA replacement have been generally consistent between observational studies that measure exposure at baseline and intervention studies that reduce exposure prospectively [12],[14],[31]. There is also evidence that former smokers reduce their risk to that of never-smokers over time [40]. Although mortality effects of other risks in our analysis have not been tested in appropriately designed and powered intervention studies, trials and observational studies provide similarly valid results on related nonfatal events for some risks, e.g., effects of BMI on incident diabetes [41],[42]. Possibly the most important case of current discrepancy between prospective observational cohorts and intervention studies is the mortality effect of high blood glucose. Prospective studies have shown relatively large associations between usual FPG and mortality [7],[43], but randomized intervention studies have shown null effects, and declines as well as increases in mortality when glucose was lowered intensively relative to those who had conventional management [44],[45]. This discrepancy may reflect the actual intervention mechanism (lifestyle versus pharmacologic treatment) or the differential effects of avoiding an increase in blood glucose versus subsequent lowering. Alternatively, blood glucose may be a partial or confounded marker of other underlying metabolic dysfunction, so that interventions targeting only glucose may be unsuccessful at ameliorating all of the observed risk. Further research is needed on the causal effects of blood glucose on mortality risk and on the role of specific lifestyle and pharmacologic interventions. Finally, there is also a need to systematically examine whether salt reduction trials with sufficiently long follow-up duration can capture the full blood pressure–lowering benefits of having maintained low salt intake throughout the life course [37].

Our results estimate the total effects of each individual risk factor. Disease-specific deaths are caused by multiple factors acting simultaneously, and hence could be prevented by intervening on single or multiple risk factors, e.g., some IHD deaths may be prevented by reducing SBP, LDL cholesterol, smoking, or combinations of these risks [46]. Further, part of the effect of one risk factor may be mediated through another, e.g., dietary factors and physical inactivity may affect IHD with part of their effect occurring by changes in BMI, blood pressure, glucose, and LDL cholesterol. Deaths attributable to multiple causally related or overlapping risk factors should not be combined by simple addition. Future analyses, both in epidemiological cohorts and at the population level, should examine the individual and combined effects of multiple exposures that affect the same diseases, including how much of the effects of lifestyle and dietary risks are mediated through metabolic factors. Finally, the effects of dietary macronutrients may vary depending on the macronutrient replacement (e.g., for PUFA; see Table 2 for details). Therefore, the interpretation of results should take such replacement issues into account.

There are a number of innovations and strengths in our analysis. This is, to our knowledge, the first population-level analysis of the mortality effects of risk factors to include a relatively large number of dietary and metabolic risk factors, and to use consistent and comparable methods. This comparative quantification helped identify the important roles of diet and physical inactivity, other lifestyle factors, and metabolic risks as preventable causes of death in the US population. Effect sizes were derived from large meta-analyses of either randomized trials or observational studies that had adjusted for important confounders. RRs from meta-analyses tend to reduce random error relative to individual studies; they may also reduce bias if the directions of bias are not the same in individual studies. We used exposure distributions and effect sizes that accounted for measurement error associated with one-off measurements to the extent possible. Our study presented deaths attributable to risk factors by age and sex, and by exposure level. The latter helped identify whether those whose exposure remains uncontrolled with current diagnosis and treatment programs versus those who are currently below clinical thresholds should be targeted for greatest effects on mortality. Finally, we quantified the sampling uncertainty of our estimates; we also analyzed how specific methods and data sources affected our quantitative results in extensive sensitivity analyses. This demonstrated that although the specific numerical results are uncertain, our overall findings on the relative mortality effects of these dietary, lifestyle, and metabolic risk factors are robust.

Population level analyses of mortality effects of risk factors such as ours are also affected by some limitations and uncertainties. First, several potentially important risk factors were considered, but could not be included because sufficient or unbiased data on their national exposure distributions and/or effects on disease-specific mortality were not available, or because the evidence on causal effects was less convincing. Second, for many risks the choice of disease outcomes and effect sizes were derived from observational studies. In such cases, whether the collectivity of evidence established a causal association had to be assessed using multiple criteria, such as those proposed by Hill [47]. In such cases, the possibility of residual confounding cannot be excluded. Our ability to account for measurement error in exposure and to correct for regression dilution bias was limited to those risk factors for which relevant data were available from epidemiological studies; for other risks, our results should be considered as conservative estimates of the effects because regression dilution bias often, although not always, leads to lower RRs in multivariate analysis [48]. RRs from meta-analyses may not be completely generalizable to population-level effects; nevertheless, such estimation is indispensable to inform policy making. More importantly, in many cases there is empirical evidence to support the proposition that proportional effects are similar across populations, e.g., Western and Asian populations [7],[20],[21].

The hazardous effects of some risk factors accumulate gradually after exposure begins and decline slowly after exposure is reduced. This is illustrated by results from trials that have lowered blood pressure and cholesterol, and from studies in which some people quit smoking [13],[40]. Time-dependence of risk may further vary by disease, e.g., the effects of tobacco smoking on lung cancer versus cardiovascular diseases [49]. Because smoking prevalence has declined in the US, the use of the smoking impact ratio (SIR) as the metric of cumulative exposure [18] may have overestimated the cardiovascular deaths attributable to smoking. However, the difference between the estimated number of deaths using this method and using the measured prevalence of current and former smoking was <14% (Table S1). The use of RRs from cohort studies that started a few decades ago may overestimate the effects of BMI on diseases such as IHD if “mediators” such as SBP and cholesterol have been lowered over time in those with high BMI [50]–[52], but underestimate the effects for other diseases such as diabetes because the current US population gained weight at younger ages than the cohort participants. Future research should attempt to investigate time-dependent effects of blood glucose, BMI, physical activity, and dietary factors, because their exposures have changed in the US over time.

The results of our analysis of dietary, lifestyle, and metabolic risk factors show that targeting a handful of risk factors has large potential to reduce mortality in the US, substantially more than the currently estimated 18,000 deaths averted annually by providing universal health insurance [53]. Global analyses also found that a relatively modest number of risk factors were responsible for a substantial proportion of mortality and disease burden in many world regions. At the same time the mix of leading risks varied across regions, as did risk factor levels in relation to economic development and urbanization [46],[54]. Therefore there is a need for national, and even subnational, analysis of the health consequences of these risks in countries at different levels of development using local exposure data [55].

The risk factors in this analysis can be influenced through both individual-level and population-wide interventions. In particular, effective interventions are available for tobacco smoking and high blood pressure, the leading two causes of mortality in the US [56]–[58]. Combinations of food industry regulation, pricing, and better information can also be effective in reducing exposure to dietary salt and trans fatty acids, especially in packaged foods and prepared meals. Despite the availability of interventions, blood pressure and tobacco smoking decline in the US have stagnated or even reversed [39],[59], and there has been a steady increase in overweight–obesity [60]. Research, implementation, monitoring, and evaluation related to interventions that reduce these modifiable risk factors should be a high priority.

## Supporting Information

Table S1 Sensitivity of results to methodological choices and data sources.
(0.08 MB DOC)

Table S2 Comparison of estimated number of deaths attributable to risk factors with those from previous studies.
(0.07 MB DOC)

## Abbreviations

BAC: blood alcohol concentration
BMI: body mass index
CI: confidence interval
FARS: Fatality Analysis Reporting System
FPG: fasting plasma glucose
HDL: high-density lipoprotein
ICD: International Classification of Diseases
IHD: ischemic heart disease
LDL: low-density lipoprotein
NCHS: National Center for Health Statistics
NESARC: National Epidemiologic Survey on Alcohol and Related Conditions
NHANES: National Health and Nutrition Examination Survey
OR: odds ratio
PAF: population attributable fraction
PUFA: polyunsaturated fatty acids
RR: relative risk
SBP: systolic blood pressure
SD: standard deviation
SFA: saturated fatty acids
TMRED: theoretical-minimum-risk exposure distribution
