# Supplementary material for: The Preventable Causes of Death in the United States: Comparative Risk Assessment of Dietary, Lifestyle, and Metabolic Risk Factors
Source: PLoS Med. 2009 Apr 28;6(4):e1000058. doi: 10.1371/journal.pmed.1000058 (PMC2667673; doi:10.1371/journal.pmed.1000058)
Supplement: Table S1 — Sensitivity of results to methodological choices and data sources. (0.08 MB DOC) [file pmed.1000058.s001.doc]

**Table S1.** Sensitivity of results to methodological choices and data sources.

| Type of sensitivity analysis | Risk factor | Main analysis | Attributable deaths in  main analysis | Sensitivity analysis | Attributable deaths in sensitivity analysis |
| --- | --- | --- | --- | --- | --- |
| Exposure metric | High cholesterol | LDL cholesterol as exposure metric | 113,000 | Total cholesterol as exposure metric ^a^ | 120,000 |
|  | Tobacco smoking | Used smoking impact ratio (SIR) as exposure metric | 467,000 | Used prevalence of current and former smoking as exposure metric | 405,000 |
|  | High blood glucose | Used fasting plasma glucose (FPG) as measured in NHANES | 190,000 | Assumed that diabetes treatment may not reduce cardiovascular disease risk, even if it reduces FPG ^b^ | 216,000 |
| The shape of the exposure distribution | Low intake of fruits and vegetables | Used normal exposure distribution | 58,000 | Used exposure distribution observed in NHANES | 56,000 |
|  | Overweight-obesity (high BMI) | Used normal exposure distribution | 216,000 | Used exposure distribution observed in NHANES | 210,000 |
|  | High blood pressure | Used normal exposure distribution | 395,000 | Used exposure distribution observed in NHANES | 419,000 |
|  | High blood glucose | Used normal exposure distribution | 190,000 | Used exposure distribution observed in NHANES | 197,000 |
|  | High LDL cholesterol | Used normal exposure distribution | 113,000 | Used exposure distribution observed in NHANES | 115,000 |
|  | Low dietary polyunsaturated fatty acids (PUFA) | Used normal exposure distribution | 15,000 | Used exposure distribution observed in NHANES | 16,000 |
| Optimal exposure distribution, theoretical-minimum-risk exposure distribution (TMRED) | High LDL cholesterol | 2.0 mmol/L as the mean of TMRED | 113,000 | 2.3 mmol/L as the mean of TMRED ^c^ | 83,000 |
|  | High LDL cholesterol | 2.0 mmol/L as the mean of TMRED | 113,000 | 1.6 mmol/L as the mean of TMRED ^d^ | 148,000 |
|  | High BMI | 21 kg/m^2^ as the mean of TMRED | 216,000 | 23 kg/m^2^ as the mean of TMRED ^e^ | 176,000 |
|  | Low dietary PUFA | 10 % of total calories from PUFA | 15,000 | 15 % of total calories from PUFA | 39,000 |
|  | High dietary salt | 0.5 g/d of sodium as the mean of TMRED | 102,000 | 1.0 g/d of sodium as the mean of TMRED | 82,000 |
|  | Alcohol use | Abstaining for the whole population as the TMRED | 27,000 ^f^ | Regular intake of the lowest average alcohol use (0-19.9 g/d for women and 0-39.9 g/d for men) for men as the TMRED | 23,000 ^f^ |
|  | Physical inactivity | Used four categories with “highly active” as the theoretical-minimum risk category | 191,000 | Three categories with “recommended-level active” as the theoretical-minimum-risk category | 129,000 |
| Disease outcomes included in the analysis | Tobacco smoking | Colorectal cancer and hypertensive disease were not included | 467,000 | Colorectal cancer and hypertensive disease were included | 485,000 |
|  | High BMI | Non-Hodgkin lymphoma was not included | 216,000 | Non-Hodgkin lymphoma was included | 224,000 |
| Effect sizes | High LDL cholesterol | Null effect on ischemic stroke in those ≥ 70 years | 113,000 | Protective effect of lower cholesterol on ischemic stroke in those ≥ 70 years | 116,000 |
|  | High dietary salt and cardiovascular diseases | Change in systolic blood pressure (SBP) per unit reduction in salt intake from He et al [1] | 102,000 | Change in SBP per unit reduction in salt intake from Law et al [2] | 194,000 |
| Adjustment for regression dilution bias as a result of one-off measurements | Physical inactivity | Did not adjust for regression dilution bias | 191,000 | Adjusted for regression dilution bias due to one-off baseline measurement of physical activity [3] | 317,000 |
| Correlation between risk factor and disease-specific mortality | High blood pressure (correlation with ischemic heart disease; IHD) | Used population attributable fraction (PAF) estimator in the Equation 1, applied to total IHD deaths | 185,000 | Estimated PAF in 3 SBP categories (≥ 140, 120-140 and < 120 mmHg) and applied to IHD deaths in each category separately ^g^ | 200,000 |
|  | Tobacco smoking (correlation with IHD) | Used PAF estimator in the Equation 1, applied to total IHD deaths | 60,000 | Estimated PAF in former and current smokers and applied to IHD deaths in each category separately ^g^ | 58,000 |
|  | High BMI (correlation with IHD) | Used PAF estimator in the Equation 1, applied to total IHD deaths | 107,000 | Estimated PAF in normal weight, overweight and obese and applied to IHD deaths in each category separately ^g^ | 85,000 ^h^ |
| Correlation between two risk factors | High blood pressure (correlation with LDL cholesterol for effect on IHD) | Used PAF estimator in the Equation 1 | 185,000 | Used partial PAF estimator stratified on LDL cholesterol levels [4] | 242,000 |

^a^ The TMRED had a mean of 3.8 and SD of 0.6 mmol/L [5].

^b^ This analysis was conducted because the most recent evidence from clinical trials showed that intensive treatment of diabetes may reduce FPG but not the risk of the outcomes caused by diabetes [6,7]. We calculated the difference between mean FPG at baseline and at the end of follow-up in the conventional treatment arm of the ADVANCE trial [6] and used it as the mean effect of treatment in diabetics. We added this mean effect to measured FPG of diabetics in NHANES to estimate their pre-treatment FPG.

^c^ Calculated as the average LDL that would lead to the 3.8 mmol/L TMRED used for total cholesterol, based on the ratio of total to LDL cholesterol in the NHANES 2003-2006 data.

^d^ Evidence from some clinical trials supports reduced risk for levels as low as 1.6 mmol/L [8,9].

^e^ Some prospective studies have found increased mortality with BMI below 23 kg/m^2^ [10,11] [12].

^f^ These analyses do not include deaths from injuries as the proportion of these deaths that are alcohol-related can not be estimated using the regular light drinkers as the minimum-risk category.

^g^ Total IHD deaths were divided across baseline risk factor categories using the same ratios as the 1992 NHANES-I mortality follow-up.

^h^ The number of attributable deaths was lower because there is negative correlation between BMI and IHD mortality.

**Reference List**

1. He FJ, MacGregor GA (2002) Effect of modest salt reduction on blood pressure: a meta-analysis of randomized trials. Implications for public health. J Hum Hypertens 16: 761-770.

2. Law MR, Frost CD, Wald NJ (1991) By how much does dietary salt reduction lower blood pressure? III--Analysis of data from trials of salt reduction. BMJ 302: 819-824.

3. Emberson JR, Whincup PH, Morris RW, Wannamethee SG, Shaper AG (2005) Lifestyle and cardiovascular disease in middle-aged British men: the effect of adjusting for within-person variation. Eur Heart J 26: 1774-1782.

4. Spiegelman D, Hertzmark E, Wand HC (2007) Point and interval estimates of partial population attributable risks in cohort studies: examples and software. Cancer Causes Control 18: 571-579.

5. Lawes CC, Vander Hoorn S, Law MR, Rodgers A (2004) High cholesterol. In: Ezzati M, Lopez AD, Murray CJ, Rodgers A, editors. Comparative quantification of health risks: Global and regional burden of disease attributable to selected major risk factors. Geneva: WHO. pp. 1651-1801.

6. Patel A, MacMahon S, Chalmers J, Neal B, Billot L, et al. (2008) Intensive blood glucose control and vascular outcomes in patients with type 2 diabetes. N Engl J Med 358: 2560-2572.

7. Gerstein HC, Miller ME, Byington RP, Goff DC, Jr., Bigger JT, et al. (2008) Effects of intensive glucose lowering in type 2 diabetes. N Engl J Med 358: 2545-2559.

8. Barter P, Rye KA (2006) Are we lowering LDL cholesterol sufficiently? Nat Clin Pract Cardiovasc Med 3: 290-291.

9. Cannon CP, Braunwald E, McCabe CH, Rader DJ, Rouleau JL, et al. (2004) Intensive versus moderate lipid lowering with statins after acute coronary syndromes. N Engl J Med 350: 1495-1504.

10. Flegal KM, Graubard BI, Williamson DF, Gail MH (2005) Excess deaths associated with underweight, overweight, and obesity. JAMA 293: 1861-1867.

11. Flegal KM, Graubard BI, Williamson DF, Gail MH (2007) Cause-specific excess deaths associated with underweight, overweight, and obesity. JAMA 298: 2028-2037.

12. Manson JE, Bassuk SS, Hu FB, Stampfer MJ, Colditz GA, Willett WC (2007) Estimating the number of deaths due to obesity: can the divergent findings be reconciled? J Womens Health (Larchmt ) 16: 168-176.
