# Supplementary material for: The Preventable Causes of Death in the United States: Comparative Risk Assessment of Dietary, Lifestyle, and Metabolic Risk Factors
Source: PLoS Med. 2009 Apr 28;6(4):e1000058. doi: 10.1371/journal.pmed.1000058 (PMC2667673; doi:10.1371/journal.pmed.1000058)
Supplement: Table S2 — Comparison of estimated number of deaths attributable to risk factors with those from previous studies. (0.07 MB DOC) [file pmed.1000058.s002.doc]

**Table S2.** Comparison of estimated number of deaths attributable to risk factors with those from previous studies ^a^.

| **Risk factor** | **Attributable deaths in this analysis** | **Selected previous study (year of estimates)** | **Attributable deaths in previous study** | **Reason(s) for differences** |
| --- | --- | --- | --- | --- |
| Tobacco smoking | 467,000 | McGinnis and Foege [1] | 400,000^*^ | - Used current and former smoker prevalence (leading to lower estimates than smoking impact ratio [SIR]). - Estimates were for 1990, when there were fewer deaths (leading to lower estimates). - Did not include diabetes as an outcome. |
|  |  | Mokdad et al [2] | 435,000 | - Used current and former smoker prevalence (leading to lower estimates than SIR). - Prevalence data were from BRFSS 2000, which are lower than those in NHANES 2003-2006 (leading to lower estimates). - Included mortality effects of environmental tobacco smoke and infant mortality due to maternal smoking (leading to higher estimates). - Did not include diabetes as an outcome. |
|  |  | Surgeon General’s report [3] | 440,000 | - Used current and former smoker prevalence (leading to lower estimates than SIR). - Included mortality effects of environmental tobacco smoke, infant mortality due to maternal smoking and deaths from cigarette-caused residential fires (leading to higher estimates). - Prevalence of smoking was from National Health Interview Survey (NHIS) 1995-1999, which are lower than those in NHANES 2003-2006 (leading to higher estimates). - Did not include diabetes as an outcome. |
|  |  | Rivara et al [4] | 412,000 | - Used current and former smoker prevalence (leading to lower estimates than SIR). - Used a projection model for the prevalence of smoking in a cohort of 18 year olds in 200 that estimates lower prevalence than the current prevalence in the same age-sex group. - Did not include diabetes as an outcome. |
|  |  | Thun et al [5] | 397,000^*^ | - Used current and former smoker prevalence (leading to lower estimates than SIR). - Estimates were for 1990, when there were fewer deaths (leading to lower estimates). - Did not include diabetes as an outcome. |
| Alcohol use | 64,000 | Mokdad et al [2] | 85,000^*^ | - Assumed all alcohol-related injuries were caused by alcohol (leading to higher estimates). - Did not consider protective effects of regular drinking on ischemic heart disease (IHD) and ischemic stroke (leading to higher estimates). |
|  |  | McGinnis and Foege [1] | 105,000^*^ | - Assumed that all alcohol-related injuries are caused by alcohol (leading to higher estimates). - Did not consider protective effects of regular drinking on IHD and ischemic stroke (leading to higher estimates). |
|  |  | Center for Disease Control and Prevention [6] | 76,000^*^ | - Assumed all alcohol-related injuries were caused by alcohol (leading to higher estimates). - Alcohol consumption was from Behavioral Risk Factor Surveillance System (BRFSS) which is a telephone survey and uses a different instrument than the one used in NESARC. The exposure estimates are not directly comparable but it is generally believed that telephone surveys may underestimate consumption [7]. - Did not consider protective effects of regular drinking on IHD and ischemic stroke (leading to higher estimates). |
| Overweight-obesity (high BMI) | 216,000 | Mokdad et al [2] | 350,000^*^ [8] | - RRs were from 6 large cohort studies that found increased risk in BMI < 23 kg/m^2^ compared to normal BMI category (23-25 kg/m^2^) (leading to higher estimates). - Used all-cause mortality (the direction and magnitude of how it affects the estimated number of attributable deaths depend on whether and how the compositions of the causes of deaths are the same or different in the cohorts used to derive RRs and the general population). - Calculated the average of attributable deaths in 1990 and 2000 to account for time lag in mortality effects of obesity (leading to lower estimates). |
|  |  | Flegal et al [9] | 163,000 for BMI ≥ 30 kg/m^2^;  - 42,000 for BMI 25-30 kg/m^2 *^ | - Used large categories of BMI (< 18.5, 18.5-25, 25-30, 30-35, ≥ 35 kg/m^2^) (generally leading to lower estimates than a continuous exposure metric because it does not incorporate the mortality effects within each BMI category). - Found protective effect on mortality in the overweight category compared to normal (leading to lower estimates; the estimated 163,000 deaths attributable to BMI ≥ 30 kg/m^2^ compared to normal weight is slightly higher than our estimate of 136,000 for the same category). ^b^ - Effects were calculated relative to a BMI reference of 18.5 to 25 kg/m^2^. The mortality affects associated with BMI ≥ 30 kg/m^2^ in our analysis were 122,000 when we used a mean BMI of 23 kg/m^2^ as our optimum in our sensitivity analysis. - Used the partial PAF estimator (leading to lower estimates in our sensitivity analysis). - Used aggregate disease categories of cardiovascular diseases, cancers and “all other causes of death” (the direction and magnitude of how it affects the estimated number of attributable deaths depend on whether and how the compositions of the causes of deaths are the same or different in the cohorts used to derive RRs and the general population). |
|  |  | Flegal et al [10] | 112,000 for BMI ≥ 30 kg/m^2^;  - 86,000 for BMI 25-30 kg/m^2 *^ | - Used large categories of BMI (< 18.5, 18.5-25, 25-30, 30-35, ≥ 35 kg/m^2^) (generally leading to lower estimates than a continuous exposure metric because it does not incorporate the mortality effects within each BMI category). - Found protective effect on mortality in the overweight category compared to normal (leading to lower estimates; the estimated 112,000 deaths attributable to BMI ≥ 30 kg/m^2^ compared to normal weight is also lower than our estimate of 136,000 for the same category). ^b^ - Effects were calculated relative to a BMI reference of 18.5 to 25 kg/m^2^. The mortality affects associated with BMI ≥ 30 kg/m^2^ in our analysis were 122,000 when we used a mean of 23 kg/m^2^ as our optimum in our sensitivity analysis (Table S3). - Used the partial PAF estimator (leading to lower estimates in our sensitivity analysis). - Used all-cause mortality (the direction and magnitude of the effect on the estimated number of attributable deaths depends on whether the compositions of the causes of deaths are the same or different in the cohorts used to derive RRs and the general population). Analysis of slightly more specific causes of death by the same authors led to a larger number of attributable deaths (see [9]). |
|  |  | Allison et al [11] | 280,000^*^ | - Exposure was from NHANES III (1988-1994) (leading to lower estimates because BMI has increased over time). - Effects were calculated relative to a BMI reference of 23-25 kg/m^2^ (leading to lower estimates). - Estimates were for 1991, when there were fewer deaths (leading to lower estimates). - Used all-cause mortality (the direction and magnitude of the effect on the estimated number of attributable deaths depends on whether the compositions of the causes of deaths are the same or different in the cohorts used to derive RRs and the general population). - RRs were derived from 6 large prospective cohort studies and were applied to exposure estimates separately. The PAFs were then simply averaged over these 6 sources (leading to higher estimates as the studies with lower sample sizes had larger RRs, These studies would have been given smaller weight if RRs from a meta-analysis were used). |
| High dietary salt | 102,000 | Havas et al 2004 [12] | 150,000^*^ | - Calculated the effect of 50% reduction in sodium intake (leading to lower estimates as halving the current 3 g/d intake levels would be substantially higher than our optimum level of 0.5 g/d). - Did not use individual-level analysis of the effect of reduction in salt intake on blood pressure (leading to higher estimates). - Effect size for salt-systolic blood pressure (SBP) relationship from a different meta-analysis (leading to lower estimates). - Decline in SBP was related to all-cause mortality, using effect sizes from Multiple Risk Factor Intervention Trial [13] (leading to higher estimates because the proportion of deaths from cardiovascular diseases has declined in the last two decades). |
| High dietary trans fatty acids | 82,000 | Mozaffarian et al [14] | 72,000 to 226,000 IHD events | - The outcome was IHD events (leading to higher estimates because not all IHD events are fatal). Applying the PAF range to IHD deaths gives attributable deaths from 30,000 to 94,000 which includes our estimate of 82,000. - Used lower current dietary trans fatty acids (leading to lower estimates). - Effects were calculated relative to 0.1 percent of calories from trans fat, which is lower than our TMRED (leading to higher estimates). |

^*^ More than 10% difference between our results and those from previous analysis.

^a^ In addition to using comparable methods, our study included a number of new metabolic and dietary risk factors that were not included in previous studies; comparisons could not be made for these risks.

^b^ The observed protective effect in the overweight category may be partly due to improved treatment for intermediate risk factors such as high blood pressure and high cholesterol in the overweight category in the US, especially in the later rounds of NHANES [15]. There is also a possibility of bias due to residual confounding by smoking, confounding by socio-economic status, diet or physical activity and existence of sub-clinical disease at baseline.

**Reference List**

1. McGinnis JM, Foege WH (1993) Actual causes of death in the United States. JAMA 270: 2207-2212.

2. Mokdad AH, Marks JS, Stroup DF, Gerberding JL (2004) Actual causes of death in the United States, 2000. JAMA 291: 1238-1245.

3. US Department of Health and Human Services (2004) The Health Consequences of Smoking: A Report of the Surgeon General.

4. Rivara FP, Ebel BE, Garrison MM, Christakis DA, Wiehe SE, Levy DT (2004) Prevention of smoking-related deaths in the United States. Am J Prev Med 27: 118-125.

5. Thun MJ, Apicella LF, Henley SJ (2000) Smoking vs other risk factors as the cause of smoking-attributable deaths: confounding in the courtroom. JAMA 284: 706-712.

6. Anon. (2004) Alcohol-attributable deaths and years of potential life lost--United States, 2001. MMWR Morb Mortal Wkly Rep 53: 866-870.

7. Rehm J, Spuhler T (1993) Measurement error in alcohol consumption: the Swiss Health Survey. Eur J Clin Nutr 47 Suppl 2: S25-S30.

8. Mokdad AH, Marks JS, Stroup DF, Gerberding JL (2005) Correction: actual causes of death in the United States, 2000. JAMA 293: 293-294.

9. Flegal KM, Graubard BI, Williamson DF, Gail MH (2007) Cause-specific excess deaths associated with underweight, overweight, and obesity. JAMA 298: 2028-2037.

10. Flegal KM, Graubard BI, Williamson DF, Gail MH (2005) Excess deaths associated with underweight, overweight, and obesity. JAMA 293: 1861-1867.

11. Allison DB, Fontaine KR, Manson JE, Stevens J, VanItallie TB (1999) Annual deaths attributable to obesity in the United States. JAMA 282: 1530-1538.

12. Havas S, Roccella EJ, Lenfant C (2004) Reducing the public health burden from elevated blood pressure levels in the United States by lowering intake of dietary sodium. Am J Public Health 94: 19-22.

13. Stamler R (1991) Implications of the INTERSALT study. Hypertension 17: I16-I20.

14. Mozaffarian D, Clarke R (2008) Quantitative effects on cardiovascular risk factors and coronary heart disease risk of replacing partially hydrogenated vegetable oils with other fats and oils. Eur J Clin Nutr in press:

15. Gregg EW, Cheng YJ, Cadwell BL, Imperatore G, Williams DE, et al. (2005) Secular trends in cardiovascular disease risk factors according to body mass index in US adults. JAMA 20;293: 1868-1874.
